# Complex Mechanisms of Antimony Genotoxicity in Budding Yeast Involves Replication and Topoisomerase I-Associated DNA Lesions, Telomere Dysfunction and Inhibition of DNA Repair

**DOI:** 10.3390/ijms22094510

**Published:** 2021-04-26

**Authors:** Ireneusz Litwin, Seweryn Mucha, Ewa Pilarczyk, Robert Wysocki, Ewa Maciaszczyk-Dziubinska

**Affiliations:** Department of Genetics and Cell Physiology, University of Wroclaw, 50-328 Wroclaw, Poland; sewermucha@gmail.com (S.M.); pilarczyk.e@gmail.com (E.P.)

**Keywords:** antimony, genotoxicity, DNA damage, DNA repair, cell cycle checkpoints

## Abstract

Antimony is a toxic metalloid with poorly understood mechanisms of toxicity and uncertain carcinogenic properties. By using a combination of genetic, biochemical and DNA damage assays, we investigated the genotoxic potential of trivalent antimony in the model organism *Saccharomyces cerevisiae*. We found that low doses of Sb(III) generate various forms of DNA damage including replication and topoisomerase I-dependent DNA lesions as well as oxidative stress and replication-independent DNA breaks accompanied by activation of DNA damage checkpoints and formation of recombination repair centers. At higher concentrations of Sb(III), moderately increased oxidative DNA damage is also observed. Consistently, base excision, DNA damage tolerance and homologous recombination repair pathways contribute to Sb(III) tolerance. In addition, we provided evidence suggesting that Sb(III) causes telomere dysfunction. Finally, we showed that Sb(III) negatively effects repair of double-strand DNA breaks and distorts actin and microtubule cytoskeleton. In sum, our results indicate that Sb(III) exhibits a significant genotoxic activity in budding yeast.

## 1. Introduction

Antimony is a toxic metalloid that ubiquitously occurs in the environment at very low concentrations [1]. However, antimony has multiple industrial applications leading to occupational exposure and environmental pollution, especially in the mining and smelting areas [2,3,4]. For instance, antimony serves as a catalyst in the manufacture of polyethylene terephthalate (PET) used to produce food containers and bottles [4]. Importantly, inappropriate storage conditions, mainly exposure to high temperature and prolonged storage, result in antimony leaching from plastic materials and contamination of drinking water and foods [5,6,7,8]. Unfortunately, accumulating evidence suggests that exposure to antimony leads to various adverse health effects in humans [2,9]. Importantly, antimony trioxide is classified as possibly carcinogenic to humans by the International Agency for Research on Cancer (IARC) and a pollutant of priority interest by the United States Environment Protection Agency (USEPA) [10,11]. On the other hand, pentavalent antimonials are the first line drugs for treatment of leishmaniasis, the neglected tropical parasitic disease affecting millions of people worldwide [12]. In addition, antimony compounds show a promising anticancer activity [13,14,15].

Although antimony becomes increasingly important for industry and medicine, the molecular mechanisms of antimony toxicity remain poorly understood but seem to be similar to those described for related metalloid arsenic. It has been demonstrated by several groups that Sb(III) and Sb(V) induce oxidative stress manifested by increased levels of reactive oxygen species (ROS), protein carbonylation, lipid peroxidation and mitochondrial dysfunction in animals and plants [16,17,18,19,20]. Sb(III) displays a high affinity to sulfhydryl groups and therefore is readily complexed in the cytoplasm by cysteine-rich peptides, like glutathione (GSH) and trypanothione (TSH), followed by sequestration of resulting Sb(III)-thiol conjugates into the intracellular compartments or out of the cell [21,22]. However, this may lead to a decrease of thiol buffering capacity and in consequence to redox imbalance as shown in *Leishmania* [16,23]. Sb(III) also contributes to depletion of GSH and TSH by inhibiting activity of glutathione reductase and trypanothione reductase, respectively [23,24]. The crystal structure of the *Leishmania* trypanothione reductase complexed with Sb(III) revealed that Sb(III) inhibits activity of this enzyme by binding to redox-active catalytic cysteine residues [25]. Moreover, Sb(III) exhibits a high affinity to a CCCH-type zinc finger protein domain by displacing Zn(II) from peptides [26,27] and probably interferes with folding of newly synthesized proteins leading to protein inactivation and/or aggregation [28].

Several reports have suggested that Sb(III) exhibits potential for genotoxicity. Although antimony compounds show no mutagenic properties in both bacterial and mammalian assays [29,30,31], in vitro evidence for Sb(III)-induced DNA damage has been demonstrated using the *Bacillus subtilis* DNA repair assay [29,32], the alkaline comet assay performed with human lymphocytes [33,34] and the γH2AX assay in human cell lines [35]. Moreover, in mammalian cells, Sb(III) increases the frequency of sister chromatid exchange [29], micronuclei formation [33,34,36,37] as well as chromosomal aberrations [30,31]. In contrast to the in vitro data, most studies in rodents did not reveal clastogenic alterations following exposure to antimony compounds [33,38,39,40]. However, increase in micronuclei formation and oxidative DNA damage was detected in cells isolated from mice treated with the Sb(V)-containing antileishmanial drug Glucantime [41,42]. One year chronical exposure to antimony trioxide via inhalation did not yield any genotoxic effects in rats, whereas in mice DNA damage was detected by the comet assay in lung tissue and a slight increase in micronuclei formation was observed in erythrocytes but not in reticulocytes [43]. One study has also demonstrated the induction of chromosomal aberrations in mouse bone marrow cells specifically after chronic exposure to Sb(III) [38]. Yet, this result failed to be reproduced in a later study [39]. Most data indicating genotoxic potential of antimony have been recently questioned due to fragmentary evidence and/or a poor quality of methodology; this led the authors to conclude that antimony compounds possess rather weak, and uncertain in vivo, genotoxic properties [44].

Furthermore, molecular mechanisms of antimony genotoxicity are poorly understood and seem to be complex. It has been suggested that antimony compounds cause indirect DNA damage as a result of the oxidative stress induction [44,45]. However, whereas two groups have reported that the use of antioxidants prevents antimony-induced DNA damage and/or cell death [18,46], one study has shown that in vitro co-incubation of lymphocytes with catalase or superoxide dismutase did not suppress Sb(III)-induced micronuclei formation [33]. Interestingly, to our knowledge the oxidative DNA damage has never been directly measured after antimony exposure. Limited data indicate that antimony exerts its genotoxic effects by inhibiting DNA repair. Two independent studies have reported that Sb(III) inhibits the repair of double-strand DNA breaks (DSBs) and UV-induced lesions in mammalian cell lines [27,47,48]. Except of single in vitro study suggesting direct binding of Sb(III) to herring sperm DNA [49], there is no hard evidence for covalent interactions of antimony compounds with DNA.

The aim of this study was to perform a comprehensive analysis of Sb(III) genotoxic potential in *Saccharomyces cerevisiae*, which proved to be an excellent model to study the mechanisms of action of various DNA damaging agents, including arsenic [50]. Importantly, genotoxicity of antimony has never been studied in this organism. Our present study demonstrates that Sb(III) generates various forms of DNA damage including DNA oxidation, replication-associated and Top1-dependent DNA lesions as well as oxidative stress-independent DNA breaks. We also provide evidence that Sb(III) interferes with telomere metabolism and DNA repair.

## 2. Results

### 2.1. BER, DDT and HR DNA Damage Repair Pathways Are Important for Sb(III) Tolerance

**Figure 1:**
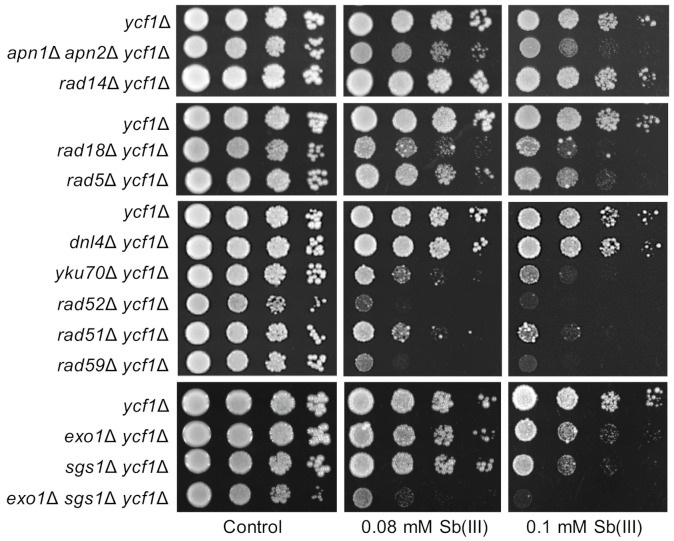
Role of DNA repair pathways in Sb(III) tolerance. Cultures of indicated mutants were serially diluted and plated on rich media in the presence or absence of Sb(III). Plates were incubated at 30 °C for 2 days and then photographed.

To investigate whether Sb(III) induces DNA damage in budding yeast, we first analyzed Sb(III) sensitivity of mutants lacking major DNA repair pathways (Figure 1). The increased sensitivity of any tested mutant to Sb(III) compared to the parental strain would be indicative of lesion generated by this metalloid. *S. cerevisiae* naturally shows high resistance to Sb(III) due the presence of the ABC transporter Ycf1, which sequestrates Sb(III) conjugated to GSH into the vacuole [51]. Therefore, to test Sb(III) at concentrations relevant for mammalian cells, we used the *ycf1*Δ mutant background in most experiments. Simultaneous deletion of *APN1* and *APN2* genes encoding for apurinic/apyrimidinic (AP) endonucleases largely inactivates base excision repair (BER) involved in the repair of chemically modified DNA bases and renders cells highly sensitive to oxidizing and alkylating agents [52]. Repair of bulky DNA adducts (e.g., UV-induced photoproducts and interstrand crosslinks) requires the nucleotide excision repair (NER) factor Rad14 that recognizes and binds damaged sites [53]. Both types of DNA damage as well as AP sites cause stalling of replication forks followed by reinitiation of DNA replication downstream of lesions that leads to the formation of ssDNA gaps behind the forks [54]. Filling of ssDNA gaps is performed by the DNA damage tolerance (DDT) pathway that operates in two modes. In S phase, ssDNA gaps are preferentially repaired by a recombination-like mechanism called template switch (TS) that depends on the Rad18 and Rad5 E3 ubiquitin ligases, recombination proteins, including Rad51 and Rad52, and nucleases such as the Sgs1-Top3-Rmi1 (STR) complex [54]. In G2 phase, ssDNA gaps can be also filled by one of translesion synthesis (TLS) low-fidelity polymerases in a Rad18-depedendent manner [55]. In budding yeast, DSBs are predominantly repaired by homologous recombination (HR) with only a minor role of non-homologous end joining (NHEJ) that depends on the yKU (Yku70-Yku80) complex and the DNA ligase IV Dnl4 [56,57].

To test the importance of DNA repair pathways in Sb(III) tolerance, mutants devoid of BER (*apn1*Δ *apn2*Δ), NER (*rad14*Δ), DDT (*rad18*Δ, *rad5*Δ, *sgs1*Δ), NHEJ (*dnl4*Δ, *yku70*Δ) and HR (*rad51*Δ, *rad52*Δ, *rad59*Δ) were plated on rich media in the presence or absence of Sb(III) (Figure 1). We found that inactivation of genes involved in BER, DDT and HR but not NER resulted in increased sensitivity of deletion mutants to Sb(III). These data suggest that Sb(III) treatment may result in chemical modifications of DNA bases that lead to replication-associated DNA damage. Higher sensitivity of *rad18*Δ to Sb(III) compared to *rad5*Δ and *sgs1*Δ indicates that both TLS and TS sub-pathways of DDT are engaged in the repair of Sb(III)-induced DNA damage. Interestingly, *rad52*Δ and *rad59*Δ mutants showed the highest sensitivity to Sb(III), whereas *rad51*Δ was moderately sensitive to Sb(III) (Figure 1). The Rad52 recombinase is involved in all known pathways of HR repair and cells lacking Rad52 are the most sensitive to DSB-inducing agents [56]. In contrast, the Rad52 paralog Rad59 plays a minor role in a Rad51 and Rad52-dependent DSB-induced recombination between sister chromatids [56] and is not involved in TS [58]. Consequently, the *rad59*Δ mutant exhibits moderate sensitivity to DSB-inducing agents compared to *rad51*Δ and *rad52*Δ [56]. On the other hand, Rad59 contributes to Rad51-independent recombination events, i.e., single-strand annealing (SSA) and break-induced replication (BIR) [59]. Higher sensitivity of *rad59*Δ to Sb(III) compared to other single DNA repair mutants suggests that Sb(III) may induce DNA lesions that are specifically repaired by Rad52/Rad59-dependent SSA and/or BIR. Repair of DSBs by recombination requires 5′ end resection catalyzed by the Exo1 and Dna2/STR (Sgs1-Top3-Rmi1) nucleases to generate 3′ ssDNA overhangs [60,61]. Interestingly, the *exo1*Δ *sgs1*Δ double mutant, which is defective in DSB resection, showed very high sensitivity to Sb(III) indicating an important role of Exo1 and Sgs1 in the repair of Sb(III)-induced DNA damage (Figure 1). We also assessed the importance of NHEJ for viability under Sb(III) exposure. Interestingly, cells lacking Yku70 but not the DNA ligase IV Dnl4, showed sensitivity to Sb(III) (Figure 1). Since both Dnl4 and the yKU complex are crucial for NHEJ, our data imply a NHEJ-independent role of yKU in tolerance to Sb(III). The yKu complex plays a key role in the maintenance of telomeric structures [62]. Thus, Sb(III) may also interfere with telomere stability.

In sum, our genetic data suggest that Sb(III) may induce several types of DNA damage, including chemical modifications of DNA bases, replication-associated DNA lesions and DSBs as well as telomere damage.

### 2.2. Sb(III) Induces DNA Damage in Yeast Cells

**Figure 2:**
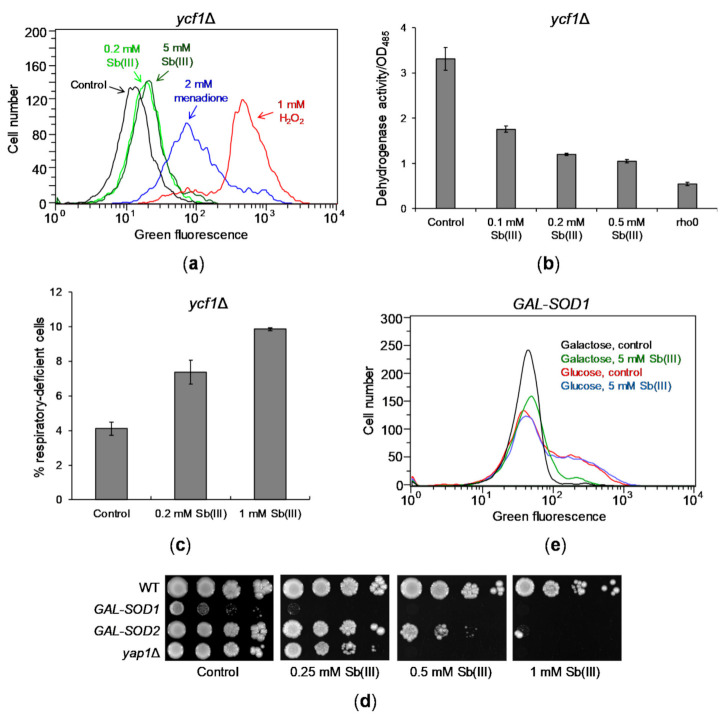
Sb(III) generates mild oxidative stress and mitochondrial dysfunction. (**a**) Sb(III) is a weak inducer of ROS production in yeast cells. The *ycf1*Δ mutant was exposed to indicated concentrations of Sb(III), menadione and H_2_O_2_ in the presence of ROS probe DHR123 for 2 h and subjected to the flow cytometry analysis. Increased levels of green fluorescence reflects formation of R123 as a result of DHR123 oxidation by ROS. Black line, untreated culture; light green line, 0.2 mM Sb(III); dark green line, 5 mM Sb(III); blue line, 2 mM menadione; red line, 1 mM H_2_O_2_. (**b**) Sb(III) impairs mitochondrial functions as measured by downregulation of dehydrogenase activity. Reduction of colorless artificial substrate triphenyltetrazolium chloride (TTC) to a pink product was used to demonstrate the activity of dehydrogenase enzymes. The *rho*^0^ mutant devoid of mitochondrial DNA was used as a positive control of respiratory defective cells. (**c**) Sb(III) elevates the rate of mitochondrial DNA loss. The *ycf1*Δ mutant was treated with indicated concentrations of Sb(III) for 3 days. The percentage of respiratory-deficient cells was determined by their inability to grow on medium with non-fermentable source of carbon. (**b**,**c**) Each bar represents the mean of four independent experiments (each with at least of 3 replicates) with standard deviations (SD). (**d**) The effect of *SOD1* and *SOD2* repression on Sb(III) tolerance. Indicated yeast strains were grown on galactose rich media to maintain expression of *SOD1* and *SOD2* and then washed before plating on rich glucose media (to repress *GAL* promoter) in the presence or absence of Sb(III). Plates were incubated at 30 °C for 3 days and then photographed. (**e**) Sb(III) does not increase production of ROS in the absence of superoxide dismutase Cu/Zn Sod1. The *GAL*-*SOD1* strain was grown on galactose (gal) or glucose (glu) and exposed to 5 mM Sb(III) for 2 h in the presence of DHR123 and analyzed by flow cytometry.

It has been previously reported that Sb(III) induces oxidative stress-derived DNA damage in mammalian and *Leishmania* cells [18,46]. Increased sensitivity of the BER-defective *apn1*Δ *apn*2Δ mutant to Sb(III) suggests that Sb(III) also induces oxidative DNA damage in *S. cerevisiae*. To test this hypothesis, we first checked whether Sb(III) increases production of ROS in yeast cells. To this end, we monitored production of green fluorescent rhodamine 123 (R123) formed as a result of ROS-mediated oxidation of nonfluorescent dihydrorhodamine 123 (DHR123) in *ycf1*Δ cells treated with Sb(III) using flow cytometry (Figure 2A). In addition, cells were exposed to 1 mM H_2_O_2_ and 2 mM menadione, superoxide-generating agent, as positive controls of oxidative stress. In contrast to menadione and H_2_O_2_ treatment, 2 h incubation with 0.2 mM Sb(III) resulted in a slight increase of green fluorescence compared to untreated culture. Also, exposure to 5 mM Sb(III) did not significantly increase formation of R123 indicating low levels of Sb(III)-induced oxidative stress (Figure 2A). As the main source of ROS are mitochondria [63,64] and heavy metals impair functionality of mitochondria leading to elevated oxidative stress [65,66,67], we investigated whether Sb(III) targets mitochondria and induces mitochondrial DNA damage. To this end, we used dehydrogenase activity assay to determine the respiratory activity of yeast cells exposed to Sb(III) [68] and found that Sb(III) substantially decreased activity of dehydrogenases suggesting Sb(III)-induced mitochondrial dysfunction (Figure 2B). Moreover, 72 h exposure to Sb(III) resulted in about 2-fold increase in formation of respiratory-deficient mutants (Figure 2C). Genetic analysis and DNA staining revealed that about 90% of Sb(III)-induced respiratory-deficient mutants exhibited loss of mitochondrial DNA. Taken together our data indicate that Sb(III) generates low levels of oxidative stress and a mild mitochondrial dysfunction in budding yeast.

However, we have previously shown that deletion of the *S*. *cerevisiae* gene *YAP1* encoding a major transcriptional activator of genes involved in maintaining redox balance causes increased sensitivity to Sb(III) (Figure 2D) [69]. This suggests that Sb(III)-induced oxidative stress might be effectively relieved by the antioxidant defense system in yeast cells. To test this assumption, we analyzed the consequence of transcriptional repression of *SOD1* and *SOD2* genes encoding superoxide dismutases. Cu/Zn Sod1 detoxifies superoxide in the cytosol and in the mitochondrial intermembrane space, whereas Mn Sod2 is exclusively localized in the mitochondrial matrix [70]. In addition, Sod1 promotes transcription of oxidative stress response genes [71]. Moreover, Sod1 plays a major role in tolerance to a wide range of ROS-generating stress conditions and depletion of Sod1 results in more severe phenotypes compared to *sod2* mutants [70,72]. Here, we found that repression of *SOD1* resulted in slow growth and high sensitivity to Sb(III), whereas depletion of *SOD2* caused moderate sensitivity to Sb(III) (Figure 2D). As expected, cells devoid of *SOD1* expression exhibited increased levels of ROS (Figure 2E). However, we did not observe further increase in ROS production in these cells during Sb(III) treatment (Figure 2E). This indicates that Sb(III) is not a potent inducer of ROS in budding yeast but may interfere with the antioxidant metabolism pathways, which are crucial for survival in the absence of superoxide dismutases, or may exert another type of stress causing additive cytotoxicity.

**Figure 3:**
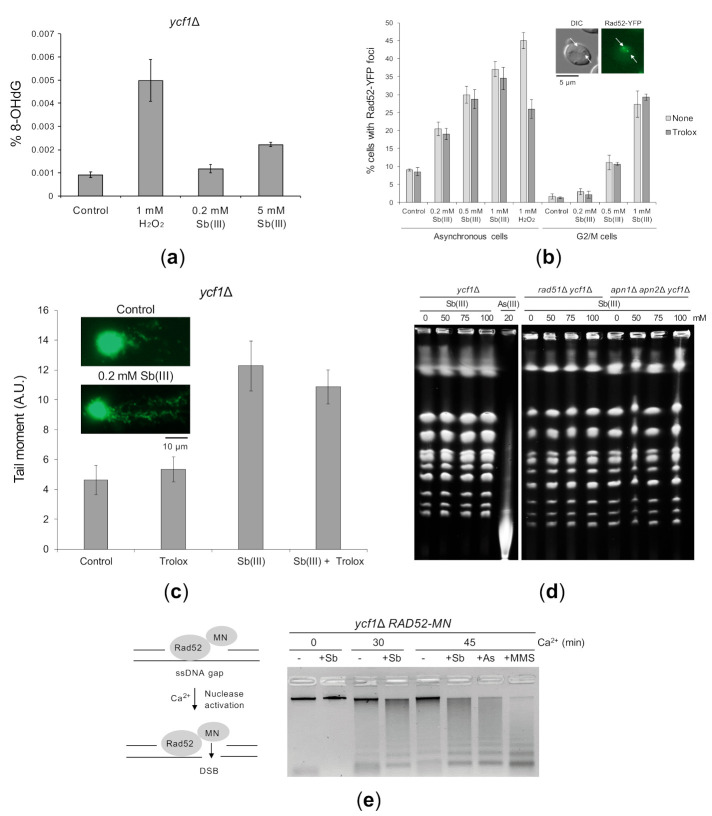
Sb(III)-induced DNA damage in yeast cells. (**a**) Sb(III) induces low levels of oxidative DNA damage. The *ycf1*Δ mutant was treated with indicated concentrations of Sb(III) or H_2_O_2_ for 2 h followed by DNA extraction and quantification of 8-hydroxy-2′-deoxyguanosine (8-OHdG) using ELISA kit. Error bars represent the mean value ± SD (*n* = 3). (**b**) Sb(III) triggers formation of Rad52-YFP nuclear foci. Asynchronous and G2/M-arrested cells were exposed to indicated concentrations of Sb(III) or H_2_O_2_ for 2 h in the absence or presence of 1 mM Trolox. Live cells were analyzed by fluorescence microscopy to visualize Rad52-YFP foci. Error bars represent the mean value ± SD (*n* = 3). Representative image of Rad52-YFP foci is shown. DIC, differential interphase contrast. Scale bar: 5 μm. (**c**) Analysis of Sb(III)-induced DNA damage by the comet assay. Exponentially growing *ycf1*Δ cells were treated with 0.2 mM Sb(III) for 2 h or mock-treated in the absence or presence of 1 mM Trolox. The tail moment was calculated based on the analysis of 200 DNA comets from three independent experiments, with at least 50 comets per experiment (mean ± SD). Representative images of DNA comets are shown. A.U., arbitrary units. Scale bar: 10 μm. (**d**) PFGE analysis of yeast chromosomes isolated from indicated strains exposed to various concentrations of Sb(III) or As(III) for 6 h or left untreated. (**e**) ChEC analysis revealed the presence of replicative DNA lesions induced by Sb(III). The *ycf1*Δ *RAD52-MN* strain was synchronized in G1 and released in S phase in the presence of 0.2 mM Sb(III), 0.2 mM As(III) or 0.05% MMS for 2 h. Before total DNA extraction, cells were permeabilized and treated with 2 mM CaCl_2_ to initiate DNA cleavage by the Rad52-MN fusion protein. Quantifications of DNA digestion are shown in Supplementary Figure S1.

To test whether Sb(III) induces oxidative stress-derived DNA damage, we first isolated genomic DNA from *ycf1*Δ cells treated with Sb(III) or H_2_O_2_ and monitored levels of 8-hydroxy-2′-deoxyguanosine (8-OHdG), which is commonly used as a marker of oxidative DNA damage [73]. We found 5-fold increase in 8-OHdG levels in the DNA isolated from *ycf1*Δ cells exposed to 1 mM H_2_O_2_ and only 2-fold increase after exposure to 5 mM Sb(III), whereas no significant increase of 8-OHdG levels was detected in the presence of 0.2 mM Sb(III) (Figure 3A). ROS-related DNA lesions also include AP sites, single-stranded DNA breaks (SSBs), replication-dependent ssDNA gaps and DSBs, which originate from closely spaced SSBs on opposite strands or a single SSB encountered by the replication fork [74]. Our genetic analysis suggested the presence of Sb(III)-induced replication-associated DNA lesions and DSBs, which require DDT and HR pathways for repair (Figure 1). To test this hypothesis, we analyzed formation of nuclear Rad52-YFP fluorescence foci that correspond to the centers of recombination repair of DSBs and replication-induced DNA lesions [75,76]. As shown in Figure 3B, Sb(III) treatment significantly increased the incidence of Rad52-YFP foci in exponentially growing cells in dose-dependent manner. We also observed Sb(III)-induced Rad52-YFP foci in cells synchronized in G2/M phase of the cell cycle, although at lower levels compared to asynchronously growing cells (Figure 3B). Importantly, induction of Rad52-YFP foci by Sb(III) was not diminished in the presence of Trolox, a water-soluble analog of α-tocopherol and powerful scavenger of ROS. In a control experiment, pretreatment with Trolox considerably reduced the incidence of H_2_O_2_–induced Rad52-YFP foci (Figure 3B). These results suggest that Sb(III) induces both replication-dependent and independent DNA lesions. Moreover, Sb(III)-induced DNA damage triggering formation of Rad52 foci is not derived from oxidative stress.

We next performed the single cell electrophoresis/alkaline comet assay that allows to detect SSBs, DSBs and alkali-labile sites in individual cells [77,78]. Alkaline-labile sites include AP sites, which are formed as result of removal of damaged bases by DNA glycosylases during BER [79]. Logarithmically growing *ycf1*Δ cells were treated with 0.2 mM Sb(III) for 2 h and then processed for the comet assay. The comet head represents the intact DNA and the comet tail is formed by the relaxed, broken DNA loops, which migrate towards the anode during electrophoresis. We used the tail moment (the tail length × % of DNA in the tail) to quantify DNA damage in yeast cells. We found that exposure to Sb(III) resulted in about 2-fold increase in the tail moment (Figure 3C). Importantly, the presence of antioxidant Trolox did not prevent accumulation of Sb(III)-induced DNA damage strongly suggesting that Sb(III) is able to generate oxidative stress-independent DNA damage (Figure 3C). However, the comet assay does not differentiate between SSBs, DSBs or AP sites. We have previously shown that exposure of yeast cells to high concentrations of As(III) results in generation of DSBs, which can be detected by pulsed field gel electrophoresis (PFGE) [50]. In addition, this technique allows to detect heat-labile DNA lesions, such as damaged bases and AP sites, which are converted to DSBs during chromosomal DNA preparation [80,81]. Thus, we isolated intact chromosomes from yeast cells exposed to high concentrations of Sb(III) (50–100 mM) and 20 mM As(III) as a positive control and subjected them to PFGE. In contrast to As(III), Sb(III) did not induce detectable DNA fragmentation (Figure 3D). To maximize our chances to visualize Sb(III)-induced DSBs or heat-labile lesions, we performed PFGE on yeast chromosomes isolated from cells devoid of recombination protein Rad51 or Apn1 and Apn2 endonucleases. However, even in the absence of HR and AP endonucleases, we failed to detect DNA fragmentation after Sb(III) exposure (Figure 3D). This suggests that either Sb(III) is not capable of inducing DSBs and heat-labile lesions or Sb(III) generates very low levels of such DNA damage, which are undetectable by PFGE.

To directly demonstrate replication-associated DNA damage triggered by Sb(III), we performed a chromatin endogenous cleavage (ChEC) analysis in *ycf1*Δ cells expressing the Rad52 protein fused at its C-terminus with the micrococcal nuclease (MN). Rad52 binds to ssDNA at resected DSBs, stalled replication forks or ssDNA gaps [76]. Recruitment of Rad52-MN to ssDNA-containing structures triggers Ca^2+^-dependent MN activation resulting in ssDNA digestion and chromosomal DNA fragmentation that can be monitored by a standard electrophoresis of genomic DNA. Importantly, as the cleavage of preexisting, resected DSBs does not enhance DNA fragmentation, the increased DNA digestion is mostly the result of Rad52-MN binding to ssDNA-containing replicative DNA lesions [76]. To test whether Sb(III) generates such structures, *ycf1*Δ *RAD52-MN* cells were synchronized in G1, released in fresh media in the presence or absence of 0.2 mM Sb(III) for 2 h. In addition, cells were exposed to 0.2 mM As(III) and 0.05% methyl methanesulfonate (MMS, DNA alkylation agent), which were shown to generate ssDNA gaps [50,76]. Next, cells were permeabilized and treated with Ca^2+^ to initiate DNA cleavage followed by genomic DNA extraction and electrophoresis (Figure 3E). In the absence of Ca^2+^, only a single high molecular band representing undigested genomic DNA was detected. In the presence of Ca^2+^, most of the DNA from untreated cells migrated as a high molecular top band with some DNA smear below. All tested compounds increased DNA digestion by Rad52-MN resulting in less intense top band with the concomitant appearance of low molecular DNA smear (Figure 3E). This result confirms that exposure to Sb(III) leads to generation of ssDNA-containing replicative DNA lesions.

### 2.3. DNA Damage Checkpoint Activation by Sb(III)

DSBs and ssDNA-containing replicative lesions induce DDR to activate cell cycle checkpoints and promote DNA repair. In yeast cells, DSB-inducing factors activate DDR in all phases of the cell cycle; however, G1 checkpoint activation is limited due to inhibition of DSB end resection by the yKU complex and low activity of cyclin-dependent kinase Cdc28 [82]. Low doses of H_2_O_2_ and MMS initiate DDR exclusively in S phase [83,84]. In the absence of Apn1 and Apn2 endonucleases, H_2_O_2_ and MMS-induced lesions are converted to ssDNA gaps and DSBs leading to activation of DDR also in G1 and G2/M [50,83,85]. In contrast, As(III), which is capable of generating DSBs in all phases of the cell cycle, activates DDR in S and G2/M but not in G1 [50]. Moreover, deletion of *YKU70* but not *APN1* and *APN2* genes triggers checkpoint activation in G1 due to resection of As(III)-induced DSBs [50]. Thus, a cell cycle phase-dependent activation of DDR may provide an additional information about a mode of action of genotoxins.

**Figure 4:**
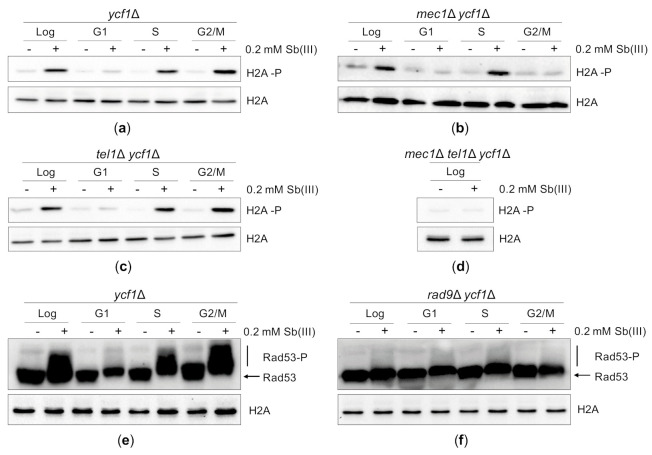
Sb(III) activates DNA damage response in budding yeast. (**a**–**d**) Sb(III) induces Mec1/Tel1-dependent histone H2A phosphorylation at S129 (H2A-P) in S and G2/M but not in G1 phase of the cell cycle. Cells were grown logarithmically (Log), synchronized and kept arrested in G1 with α-factor (G1), synchronized in G1 and released from the α-factor block to allow DNA replication (S) or synchronized and kept arrested in G2/M with nocodazole (G2/M) followed by treatment with 0.2 mM Sb(III) for 2 h or mock-treatment. Next, total protein extracts were prepared and analyzed by western blot using anti-phospho H2A (S129) and anti-H2A antibodies as a loading control. The lethality of *mec1*Δ mutation was suppressed by deletion of the *SML1* gene encoding a ribonucleotide reductase inhibitor. (**e**,**f**) Sb(III) triggers Rad9-dependent hyperphosphorylation of Rad53 (Rad53-P). The *ycf1*Δ and *rad9*Δ *ycf1*Δ cultures were prepared and treated as described above. Western blot analysis was performed with anti-Rad53 and anti-H2A antibodies as a loading control (source data are shown in Supplementary Figure S2).

To investigate whether Sb(III) triggers DDR in budding yeast, we monitored histone H2A phosphorylation at S129 (H2A-P), which is an early event in the DDR activation cascade [82]. Because H2A-P is enriched near DSBs, stalled replication forks and ssDNA gaps generated behind the forks, it is considered as a sensitive marker of DNA damage [86,87,88,89]. We also asked whether Sb(III) activates DDR in all phases of cell cycle or only during DNA synthesis. To test this, asynchronously growing or G1-, S- and G2/M-synchronized *ycf1*Δ cells were exposed to 0.2 mM Sb(III) for 2 h followed by western blot analysis of H2A-P levels. Consistently with the Rad52-YFP foci data (Figure 3B), Sb(III)-induced H2A-P was evident in asynchronously growing cells as well as in S and G2/M-synchronized cells supporting the notion that Sb(III) causes both replication-dependent and independent DNA damage (Figure 4A). However, no H2A-P signal was detected in G1-synchronized cells treated with Sb(III) as it was previously observed for As(III) [50].

DNA damage-induced phosphorylation of histone H2A at S129 is catalyzed by two DNA damage sensing kinases, Mec1 and Tel1, which belong to the phosphoinositide 3-kinase-related kinase family [86,87,88]. In yeast, Mec1 is the major DNA damage sensor kinase, which contributes to DDR activation in all phases of the cell cycle. Mec1 forms a heterodimer with the Ddc2 protein, which facilitates binding of the Mec1-Ddc2 complex to replication protein A (RPA)-coated ssDNA regions (i.e., resected DSBs, stalled replication forks, ssDNA gaps generated during replication of damaged DNA and unprotected telomeres) [88,89,90,91,92]. Tel1 is recruited to unprocessed ends of DSBs via interaction with the Mre11-Rad50-Xrs2 (MRX) complex and marginally contributes to DDR activation in G1 and S phase but not in G2/M [92,93,94]. To determine the role of Mec1 and Tel1 in Sb(III)-induced activation of DDR, we investigated levels of H2A-P in cells lacking Mec1 and Tel1 in the *ycf1*Δ background (Figure 4B–D). We found that in asynchronously growing cultures both sensor kinases contributed to H2A phosphorylation in response to Sb(III) treatment as the double *mec1*Δ *tel1*Δ mutant, but not single *mec1*Δ and *tel1*Δ mutants, exhibited loss of Sb(III)-induced H2A phosphorylation (Figure 4D). However, in G2/M cells H2A phosphorylation was fully dependent on Mec1 (Figure 4B), whereas in S phase H2A phosphorylation was triggered by both Mec1 and Tel1 (Figure 4C).

In budding yeast, H2A-P in concert with histone H3 methylated at K79 facilitates recruitment of the Rad9 adaptor protein to the chromatin near the sites of DNA damage [94,95,96]. Upon Mec1/Tel1-dependent hyperphosphorylation, Rad9 serves as a scaffold for the primary DDR effector kinase Rad53 to enable its phosphorylation by Mec1/Tel1 and subsequent *trans*-autophosphorylation resulting in full activation of the Rad53 kinase activity [97,98,99]. To investigate whether DNA damage generated by Sb(III) induces Rad53 activation, we monitored hyperphosphorylation levels of Rad53 by western blot. Similarly to H2A-P, Sb(III) triggered Rad53 phosphorylation in S and G2/M phase but not in G1 (Figure 4E). Moreover, in the absence of Rad9 no hyperphosphorylated forms of Rad53 were detected (Figure 4F). Taken together, our data indicate that Sb(III) activates the canonical Mec1/Tel1-Rad9-Rad53-dependent DDR pathway both in S and G2/M phase suggesting that Sb(III) induces not only replication-associated DNA lesions but also DSBs.

**Figure 5:**
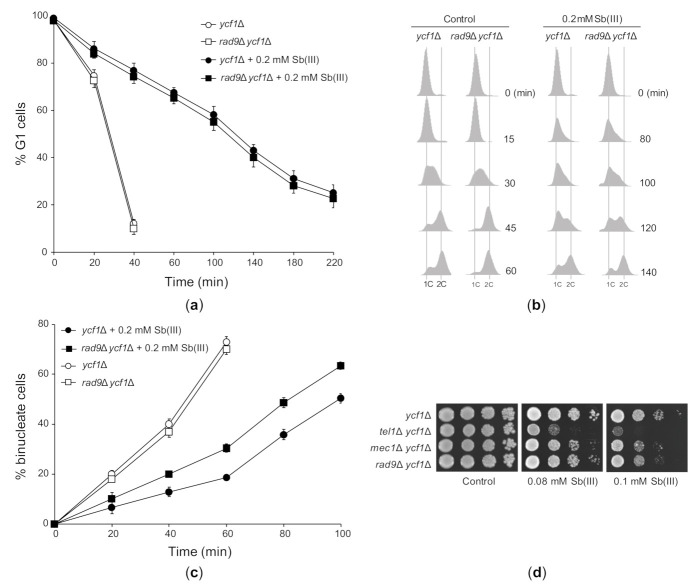
The effect of Sb(III) on cell cycle progression and the role of DNA damage checkpoint pathway in Sb(III) tolerance. (**a**) Sb(III) does not trigger checkpoint-dependent G1 delay. The *ycf1*Δ and *rad9*Δ *ycf1*Δ strains were synchronized in G1 with α-factor, washed and released in the presence or absence of 0.2 mM Sb(III). Percentage of cells arrested in G1 was determined by the α-factor-nocodazole trap assay. (**b**) Sb(III) slows S phase progression, partially in a Rad9-dependent manner. Cells were prepared and treated as described in (**a**). DNA content was determined by flow cytometry. 1C, DNA content. (**c**) Sb(III)-induced G2/M delay partially depends on Rad9. Cells were arrested at G2/M boundary with nocodazole and then released in the presence or absence of 0.2 mM Sb(III). Percentage of binucleate (post-mitotic) cells was determined by fluorescence microscopy. (**d**) Sensitivity of DNA damage checkpoint mutants to Sb(III). Cultures of indicated mutants were serially diluted and plated on rich media in the presence or absence of Sb(III). Plates were incubated at 30 °C for 2 days and then photographed.

Next, we investigated the effect of Sb(III) on cell cycle progression in *ycf1*Δ and the checkpoint-defective *rad9*Δ *ycf1*Δ mutant. In agreement with the lack of DDR activation in G1 (Figure 4A–F), deletion of *RAD9* had no impact on the dynamics of G1/S transition in the presence of Sb(III) (Figure 5A). As excepted, *rad9*Δ *ycf1*Δ cells progressed faster through S phase compared to *ycf1*Δ cells (Figure 5B) and showed partial defect in G2/M arrest (Figure 5C). However, both *ycf1*Δ and *rad9*Δ *ycf1*Δ cells also showed checkpoint-independent delays in all phases of the cell cycle probably due to cytotoxic/proteotoxic effects of Sb(III) (Figure 5A–C). Finally, we analyzed whether DDR activation is important for tolerance to Sb(III). All checkpoint defective mutants showed increased sensitivity to Sb(III) (Figure 5D). Surprisingly, the *tel1*Δ mutant was more sensitive to Sb(III) compared to single *mec1*Δ and *rad9*Δ mutants, which displayed stronger defects in DDR activation compared to *tel1*Δ (Figure 4). This strongly suggests that Tel1 also plays a checkpoint-independent role in Sb(III) tolerance.

**Figure 6:**
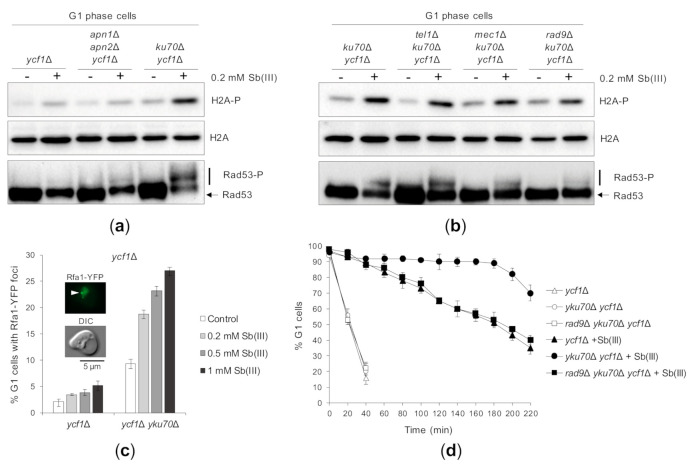
The yKu complex suppresses Sb(III)-induced DNA damage checkpoint activation in G1. (**a**,**b**) Deletion of *YKU70* results in Mec1/Tel1/Rad9-dependent G1 checkpoint activation in the presence of Sb(III). Indicated strains were synchronized in G1 with α-factor and then exposed to 0.2 mM Sb(III) for 2 h or left untreated. Next, total protein extracts were prepared and analyzed by western blot using anti-phospho H2A (S129), anti-Rad53 and anti-H2A antibodies as a loading control (source data are shown in Supplementary Figure S3). The lethality of *mec1*Δ mutation was suppressed by deletion of the *SML1* gene. (**c**) Sb(III) triggers formation of Rfa1-YFP nuclear foci in G1 cells lacking *YKU70*. G1-arrested *ycf1*Δ and *yku70*Δ *ycf1*Δ cells were exposed to indicated concentrations of Sb(III) for 2 h or left untreated. Live cells were analyzed by fluorescence microscopy to visualize Rfa1-YFP foci. Error bars represent the mean value ± SD (*n* = 3). Representative image of Rfa1-YFP foci is shown. DIC, differential interphase contrast. Scale bar: 5 μm. (**d**) Sb(III) induces Rad9-dependent G1 delay in the absence of *YKU70*. The *ycf1*Δ and *rad9*Δ *ycf1*Δ strains were synchronized in G1 with α-factor, washed and released fresh media in the presence or absence of 0.2 mM Sb(III). Percentage of cells arrested in G1 was determined by the α-factor-nocodazole trap assay.

Finally, we asked whether Sb(III)-induced DNA lesions are able to trigger DDR activation in G1-synchronized *ycf1*Δ cells lacking AP endonucleases or the yKU complex. Similarly to As(III) [50], deletion of *YKU70* but not *APN1* and *APN2* genes strongly induced phosphorylation of H2A and Rad53 (Figure 6A). Both sensor kinases Tel1 and Mec1 and the Rad9 adaptor contributed to G1 DDR activation in the *ycf1*Δ *yku70*Δ mutant (Figure 6B). Lack of H2A and Rad53 phosphorylation in the *apn1*Δ *apn2*Δ *ycf1*Δ mutant indicates that Sb(III) does not trigger high levels of closely spaced DNA lesions that could be converted to DSBs in the absence of Apn1/2. On the other hand, DDR activation by Sb(III) in the *ycf1*Δ *yku70*Δ mutant suggests that Sb(III) is able to generate low levels of DSBs, which are checkpoint-blind in G1 due the inhibitory action of the yKu complex. It is well documented that in G1 cells binding of the yKu complex to DSB ends limits the recruitment of the MRX complex and blocks resection of DSB ends [100,101,102]. Involvement of the ssDNA sensor Mec1 in G1 checkpoint activation in Sb(III)-treated *ycf1*Δ *yku70*Δ cells also implies that Sb(III) generates DSBs, which undergo resection in the absence of the yKu complex. The ssDNA regions formed as a result of DSB resection are coated by the RPA complex composed of three Rfa1-3 subunits and can be detected using the Rfa1-YFP fusion protein, which forms nuclear fluorescent foci corresponding to the sites of DSB repair [75,101]. Consistently, we found a 2–3 fold increase in the number of G1-arrested *ycf1*Δ *yku70*Δ cells exhibiting the presence of Rfa1-YFP foci after Sb(III) exposure (Figure 6C). Finally, we analyzed the effect of G1 checkpoint activation on dynamics of the G1/S transition in cells devoid of the yKU complex during Sb(III) treatment. We found that the *ycf1*Δ *yku70*Δ cells remained arrested in G1 much longer compared to *ycf1*Δ cells (Figure 6D). Importantly, deletion of *RAD9* in the *yku70*Δ *ycf1*Δ background suppressed DNA damage checkpoint -dependent G1 delay (Figure 6D). These results further support the notion that Sb(III) induces DNA lesions in all phases of the cell cycle, probably also in the form of DSBs.

### 2.4. Sb(III) Interferes with Telomere Maintenance

**Figure 7:**
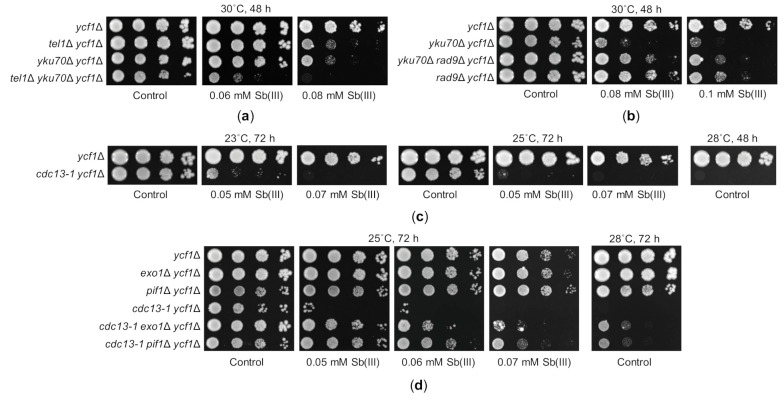
Sb(III) sensitivity of telomere maintenance mutants is suppressed by either checkpoint inactivation or preventing resection of chromosome ends. (**a**) Genetic interaction between *tel1*Δ and *yku70*Δ in the presence of Sb(III). (**b**) Deletion of *RAD9* checkpoint gene suppresses sensitivity of *yku70*Δ to Sb(III). (**c**) The *cdc13-1* mutation renders yeast cells hypersensitive to Sb(III) at both permissive (23 °C) and semi-permissive (25 °C) temperature. (**d**) Sb(III) sensitivity of *cdc13-1 ycf1*Δ cells is reversed by concomitant deletion of *EXO1* or *PIF1*. (**a**–**d**) Exponentially growing cultures were serially diluted and spotted onto YPD plates with or without Sb(III).

Telomeres are nucleoprotein structures that protect the ends of eukaryotic linear chromosomes from being recognized and processed as a DSB. Yeast telomeres consist of about 300 C1-3A/TG1-3 telomeric repeats that end with single-stranded 3′ G-rich overhang as well as subtelomeric sequences including 0–4 repeats of Y’ elements and a varied number of X elements that are highly heterogeneous in sequence and size [62,103]. Subtelomeric and telomeric regions are bound by several proteins that regulate telomere length and prevent chromosome ends from resection, DDR activation and usage as substrates for DSB repair pathways. Intriguingly, some proteins involved in DDR and NHEJ are also required for telomere homeostasis. It has been demonstrated that the DNA damage sensing checkpoint kinase Tel1 preferentially binds to short telomeres through the interaction with the Xrs2 subunit of the MRX complex, which initiates DSB resection elsewhere in the genome [104,105,106]. This supports recruitment of telomerase—a ribonucleoprotein that adds telomeric repeats to the 3′ end of telomeres. Interestingly, although Mec1 is the master regulator of DDR, it has a minor role in telomere maintenance even in *tel1*Δ cells bearing short telomeres [107]. Also, the yKU complex, which is crucial for NHEJ, plays an important role in telomere protection by preventing resection of telomeres by nucleases [108,109]. Disruption of any of the yKU subunits results in telomere degradation and shortening but no DDR activation leading to G2/M growth arrest unless *yku70*Δ or *yku80*Δ cells are cultured at 37 °C [108,109,110]. Surprisingly, we found that *tel1*Δ and *yku70*Δ mutations caused higher sensitivity to Sb(III) than other DNA damage checkpoint (*mec1*Δ and *rad9*Δ) and NHEJ (*dnl4*Δ) mutations, respectively (Figure 1,5D), whereas the double *tel1*Δ *yku70*Δ mutant was hypersensitive to Sb(III) (Figure 7A). Sensitivity of *yku70*Δ to elevated temperature due to increased levels of ssDNA at telomeres and checkpoint activation can be suppressed either by deletion of genes encoding DNA damage checkpoint proteins (e.g., Mec1 or Rad9) or the Exo1 nuclease and the Pif1 helicase involved in 5′ to 3′ resection of unprotected chromosome ends [110,111]. Similarly, we found that Sb(III) sensitivity of *yku70*Δ was also suppressed by deletion of *RAD9* suggesting enhanced erosion of telomeres leading to checkpoint-dependent growth arrest under Sb(III) stress (Figure 7B).

The single-stranded G-tails of telomeres are covered by the telomere-specific RPA-like Cdc1-Stn1-Ten1 (CST) complex, which serves as a telomere capping protein and telomerase regulator [62,103]. The temperature-sensitive *cdc13-1* mutant exhibits telomere elongation, extensive telomere resection and Mec1/Rad9-dependent DDR activation resulting in permanent G2/M arrest and cell death at the restrictive conditions [112,113,114,115]. To test the role of telomere-specific protein in Sb(III) tolerance, we introduced the *cdc13-1* mutation in the *ycf1*Δ background and checked Sb(III) sensitivity of the resulting double mutant compared to *ycf1*Δ cells. Interestingly, we found that *cdc13-1 ycf1*Δ cells exhibited a strong growth arrest in the presence of low concentrations of Sb(III) at both permissive (23 °C) and semi-permissive (25 °C) temperature (Figure 7C). This is strikingly similar to that observed for the restrictive temperature (28 °C or higher) suggesting that Sb(III) imitates high temperature conditions leading to inactivation of the Cdc13-1 variant protein. Similar to *yku70*Δ cells, the temperature sensitivity of the *cdc13-1* mutant can be suppressed by either deleting DNA damage checkpoint genes or inactivation of the Pif1 helicase or the Exo1 nuclease [110,114]. Consistently, *exo1*Δ and *pif1*Δ mutations significantly restored the growth of *cdc13-1 ycf1*Δ cells in the presence of Sb(III) (Figure 7D). These results suggest that Sb(III) accelerates telomere uncapping and resection in *cdc13-1* cells at non-restrictive conditions. Importantly, growth inhibition of *cdc13-1* cells at low temperature is specific for Sb(III) and related metalloid As(III) as no such effect was observed in the presence of cadmium, H_2_O_2_ or several DNA damaging agents tested (supplementary Figure S4). In sum, our genetic data indicate that Sb(III) treatment exacerbates telomere dysfunction in cells with compromised telomere homeostasis.

**Figure 8:**
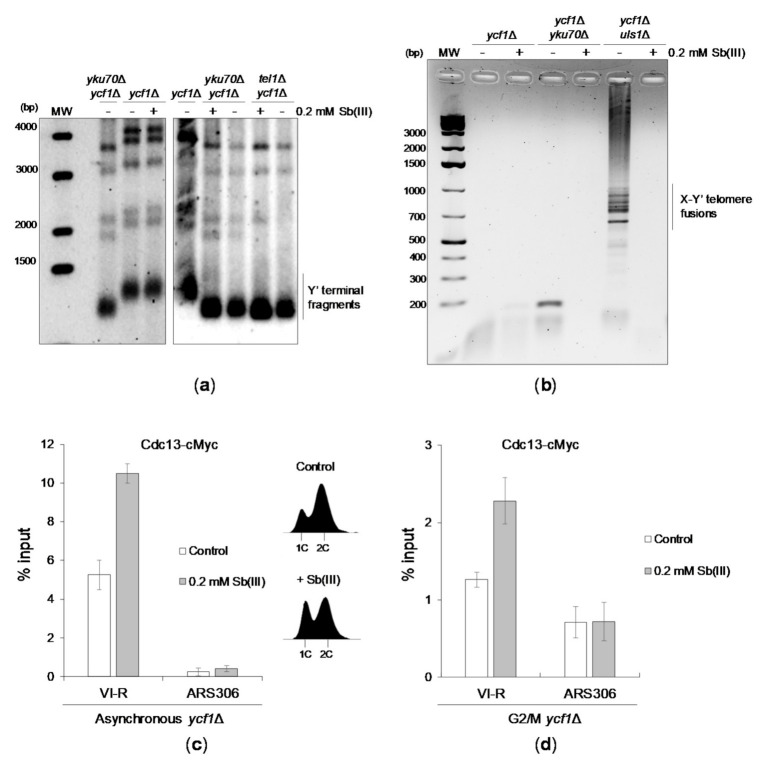
The effect of Sb(III) on telomere homeostasis. (**a**) Sb(III) does not alter telomere length in yeast cells. Cells of indicated strains were cultivated for six days in the presence or absence of Sb(III). Afterwards, genomic DNA was isolated, digested with *Xho*I and analyzed by Southern blot using a probe detecting Y’ telomere repeats. (**b**) Sb(III) inhibits telomere fusions in cells lacking Uls1. Indicated strains were cultivated in the presence or absence of 0.2 mM Sb(III) for 6 days followed by genomic DNA preparation and PCR analysis of fusions between X and Y’-only telomeres using a pair of primers specific for X and Y’ subtelomeric regions. (**c**,**d**) Cdc13 association with telomeric DNA is increased in the presence of Sb(III). The telomere VI-R DNA binding of cMyc-tagged Cdc13 in *ycf1*Δ cells treated or not treated with 0.2 mM Sb(III) for 2 h was analyzed by ChIP performed with anti-Myc antibodies followed by qPCR. ARS306 locus was used as a control for the Cdc13-free internal chromosome region. The % input value represents the enrichment of Cdc13-cMyc protein at the specific locus and is normalized to the *ACT1* reference gene. Error bars are standard deviations from two independent experiments. DNA content analysis of asynchronous cultures used for ChIP-qPCR is embedded in panel C.

Next, we investigated whether Sb(III) treatment results in telomere dysfunction manifested by changes in telomere length and/or fusions. Southern blot analysis of telomeric regions of the *ycf1*Δ mutant revealed no detectable effect of Sb(III) on telomere length (Figure 8A). As reported previously [113,116,117], *yku70*Δ and *tel1*Δ mutants exhibited short telomeres. However, no further changes in telomere length was observed upon Sb(III) treatment (Figure 8A). We next sought to determine whether Sb(III) triggers telomere fusions. To test this, exponentially growing cells in *ycf1*Δ background were grown for 6 days to reach stationary phase in the presence or absence of Sb(III). Then, genomic DNA was isolated and used as a template for PCR amplification of fusions between Y′ and X-only telomeres, which can be visualized as a ladder of bands in agarose gel after electrophoresis. Similar to previous observations [118,119], no telomere fusions were observed in NHEJ-deficient (*yku70*Δ *ycf1*Δ) cells, whereas high levels of telomere fusions were present in cells lacking the SUMO-dependent ubiquitin ligase and DNA translocase Uls1 (Figure 8B). Telomere fusions were not detected in both untreated and Sb(III)-treated *ycf1*Δ cells. Surprisingly, the presence of Sb(III) blocked ligation of telomeres in *uls1*Δ cells suggesting Sb(III)-induced inhibition of NHEJ (Figure 8B).

Finally, we investigated whether Sb(III) treatment leads to increased telomeric DNA association of Cdc13 using a chromatin immunoprecipitation-quantitative PCR (ChIP-qPCR) assay. Cdc13 association with telomeric DNA fluctuates throughout the cell cycle and peaks at the end of S phase followed by a significant decrease in G2/M that corresponds to changes of single-stranded G-tail length [104]. We found that both asynchronous cells and G2/M-arrested *ycf1*Δ cells treated with Sb(III) exhibited about 2-fold increase of telomere-bound Cdc13 (Figure 8C,D). Importantly, elevated association of Cdc13 with telomeric DNA in asynchronous cultures exposed to Sb(III) was not the result of increased content of S phase cells (Figure 8C). These data further supports the notion that Sb(III) impairs telomere homeostasis.

### 2.5. Sb(III) Triggers Top1-Induced DNA Damage

**Figure 9:**
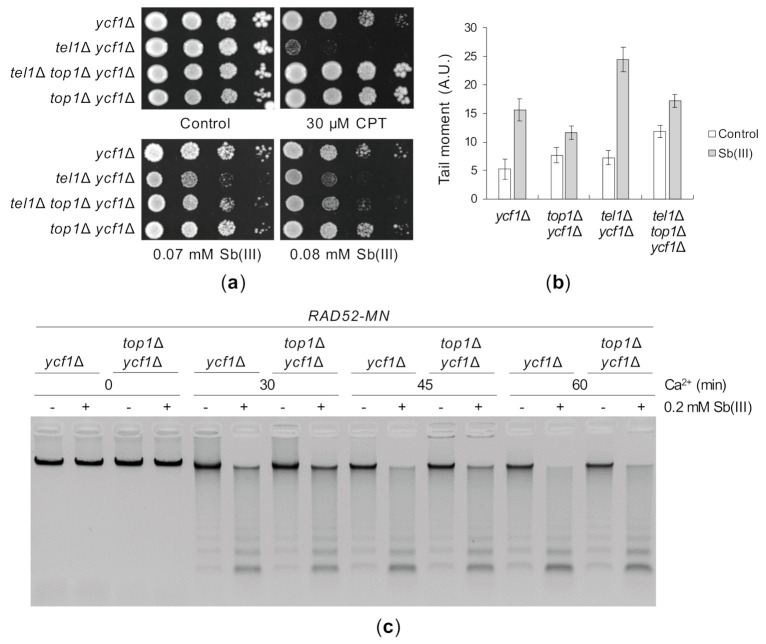
Sb(III) triggers Top1-dependent DNA lesions. (**a**) The lack of Top1 slightly improves the growth of cells with the *tel1*Δ mutation in the presence of Sb(III). Exponentially growing cell cultures were serially diluted and spotted onto YPD plates with or without indicated compounds and incubated at 30 °C for two days. (**b**) Sb(III)-induced DNA breaks partially depends on the activity of Top1. Exponentially growing cells were treated with 0.2 mM Sb(III) for 2 h or mock-treated and analyzed by the comet assay as described in Figure 3C. A.U., arbitrary units. (**c**) Deletion of *TOP1* decreases levels of Sb(III)-induced replicative DNA lesions. The presence of ssDNA-containing DNA lesions were assessed by ChEC as described in Figure 3E.

Yeast cells lacking Tel1 are not sensitive to DNA damaging agents like MMS, radiomimetic antibiotic phleomycin (PM) or irradiation, consistent with a minor role of Tel1 in DNA damage checkpoint activation in *S*. *cerevisiae* [82]. However, the *tel1*Δ mutant exhibits increased sensitivity to camptothecin (CPT), an inhibitor of the topoisomerase I Top1 [120]. It has been recently revealed that Tel1 plays a specific role in protection of reversed replication forks induced by Top1 poisoning and thus CPT sensitivity of *tel1*Δ cells is fully suppressed by *TOP1* deletion [121]. Interestingly, arsenic trioxide has been reported to induce topoisomerase I-DNA complexes via generation of oxidative DNA damage in human NB4 cell line [122]. To test whether Sb(III) impairs Top1 function leading to replication-associated lesions, we deleted the *TOP1* gene in *ycf1*Δ and *tel1*Δ *ycf1*Δ backgrounds and analyzed the growth of resulting mutants in the presence of Sb(III). As previously demonstrated, the *top1*Δ deletion fully suppressed CPT sensitivity of *tel1*Δ *ycf1*Δ cells, whereas the triple *tel1*Δ *top1*Δ *ycf1*Δ showed only a slight increase in Sb(III) resistance compared to *tel1*Δ *ycf11*Δ cells (Figure 9A). Moreover, in contrast to that observed during CPT poisoning [104], deletion of *TEL1* did not trigger Mec1-dependent checkpoint hyperactivation in the presence of Sb(III) (Figure 4). However, the lack of full suppression of *tel1*Δ sensitivity to Sb(III) by *top1*Δ may be due to additional checkpoint and telomere functions of Tel1 during Sb(III) stress (Figure 4 and Figure 7). Nevertheless, cells with deletion of the *TOP1* gene accumulated considerably less DNA breaks and ssDNA regions compared to cells expressing Top1 (Figure 9B,C). In sum, our data suggest that a subset of Sb(III)-induced DNA damage results from Top1-induced DNA lesions.

### 2.6. Sb(III) Impairs the Repair of DSBs

**Figure 10:**
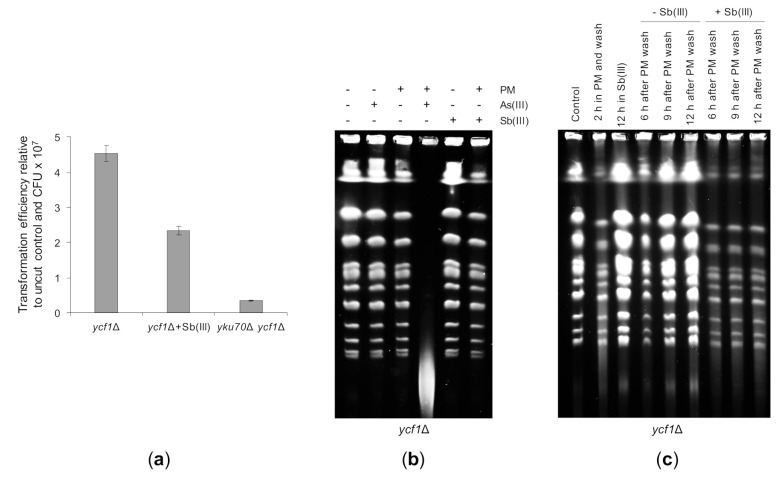
The repair of DBSs is negatively affected by Sb(III). (**a**) Sb(III) decreases the ability of yeast cells to repair cohesive DSBs by NHEJ. Indicated strains were transformed in parallel with *Pst*I-cleaved YCplac111 and supercoiled YCplac111. In addition, *ycf1*Δ cells were exposed to 0.2 mM Sb(III) for two hours, washed and used for transformation with the same set of plasmids. Transformation efficiency was expressed as the ratio of colonies obtained with the linear plasmid divided by colonies obtained with the uncut plasmid normalized to the number of viable cells used for transformation. Each bar represents the mean of four independent experiments with SD. (**b**) Sb(III) does not enhance genotoxicity of phleomycin (PM). The *ycf1*Δ mutant was exposed to 10 μg/mL PM, 5 mM As(III) or 5 mM Sb(III) for 6 h followed by isolation of genomic DNA and PGFE analysis. (**c**) Sb(III) inhibits the repair of PM-induced DSBs. The *ycf1*Δ mutant was exposed to 35 μg/mL PM for 2 h, washed and released in the presence or absence of 0.2 mM Sb(III) for up to 12 h. Cells were also left untreated or cultivated in the presence of 0.2 mM Sb(III) for 12 h. At indicated time-points, genomic DNA was prepared and analyzed by PGFE.

Lack of telomere fusions in the *uls1*Δ mutant treated with Sb(III) led to the conclusion that Sb(III) may inhibit NHEJ (Figure 8B). To test this assumption directly, we assessed NHEJ by the transformation-based plasmid rejoining assay [116]. In this assay, an yeast/*Escherichia coli* shuttle plasmid is linearized by a restriction enzyme in a region lacking homology to the yeast genome and then introduced into yeast cells by transformation. Since propagation of the plasmid requires its recircularization, transformation efficiency obtained with the linear plasmid relative to the uncut plasmid control reflects the ability of an yeast strain to repair DSB by NHEJ. The single *ycf1*Δ mutant and the double *ycf1*Δ *yku70*Δ mutant were transformed with the *Pst*I linearized or supercoiled YCplac111 plasmid. In parallel, *ycf1*Δ cells were exposed to 0.2 mM Sb(III) for 2 h prior transformation. Due to cytotoxicity of Sb(III), transformation efficiency was also normalized to the number of viable cells used for transformation expressed as colony forming units (CFU). As expected, the untreated *ycf1*Δ cells efficiently repaired the linearized plasmid, whereas the NHEJ-deficient *ycf1*Δ *yku70*Δ mutant was defective in plasmid recircularization (Figure 10A). Importantly, Sb(III)-treated *ycf1*Δ cells showed a 2-fold decrease in plasmid end-joining efficiency (Figure 10A). This result therefore supports the notion that Sb(III) inhibits DNA repair by NHEJ.

Next, we tested the effect of Sb(III) on the repair of PM-induced DSBs. We have previously shown that co-treatment of yeast cells with low doses of As(III) and PM results in massive fragmentation of chromosomes that can be visualized by PFGE as a low-molecular smear with concomitant disappearance of chromosome bands [50] (Figure 10B). Thus, we first checked whether Sb(III) enhances genotoxicity of PM. In contrast to As(III), combined treatment with PM and Sb(III) did not lead to fragmentation of chromosomes (Figure 10B). Next, we exposed yeast cells to a high dose of PM to induce DSBs and then washed the cells from PM to allow repair. In parallel, cells were released in the presence of Sb(III) to investigate its effect on the efficiency of DSB repair. PFGE analysis revealed that exposition of cells to PM for 2 h resulted in high and low molecular DNA smear and partial disappearance of chromosome bands (Figure 10C). After 9 h from wash reappearance of strong chromosome bands was observed indicating the completion of DSB repair. Importantly, in the presence of Sb(III) yeast cells failed to reconstitute chromosomes (Figure 10C). As chromosomal DSBs are predominantly repaired by HR in yeast cells [56], our data suggest that Sb(III) also impairs DSB repair through HR.

### 2.7. Sb(III) Distorts Actin and Tubulin Filaments

**Figure 11:**
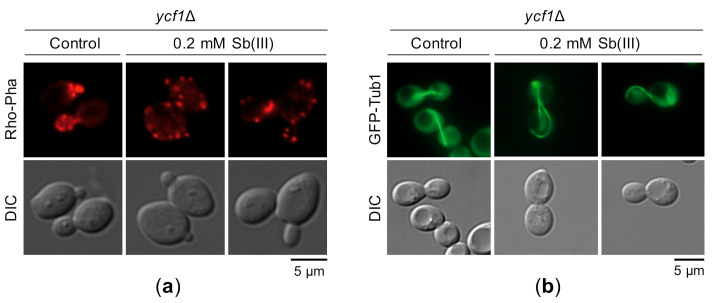
Sb(III) targets actin and microtubule cytoskeleton. (**a**) Exponentially growing *ycf1*Δ cells were exposed to 0.2 mM Sb(III) for 2 h or left untreated and then chemically fixed for actin staining with rhodamine-phalloidin. (**b**) The *ycf1*Δ mutant expressing chromosomally encoded α-tubulin fused to GFP (GFP-Tub1) was grown to log phase and then treated with 0.2 mM Sb(III) for 2 h or mock-treated. Next, viable cells were analyzed by fluorescence microscopy to visualize microtubules. Scale bars: 5 μm.

Previous reports have demonstrated that As(III) targets actin and microtubule cytoskeleton by inhibiting the chaperonin complex TRiC and the GIM/prefoldin complex required for folding of actin, tubulin and other proteins [123,124]. Interestingly, it has been shown that inhibition of actin polymerization impairs local chromosome movements correlated with reduced efficiency of HR-mediated DNA repair [125]. Based on these findings, we hypothesized that Sb(III) may also interfere with cytoskeleton organization leading to DNA repair inhibition and possibly other genotoxic effects. Indeed, Sb(III) treatment resulted in distortion of the actin filaments. In untreated *ycf1*Δ cells, cortical actin patches were concentrated in the emerging buds, whereas in the presence of Sb(III) actin patches were evenly distributed between the mother cell and the bud indicating loss of cell cycle-regulated polarization of the actin cytoskeleton (Figure 11a). Similar to As(III), the presence of Sb(III) also strongly affected morphology of microtubule filaments (Figure 11b).

## 3. Discussion

Arsenic and antimony are highly toxic metalloids showing similar modes of biological activity. Arsenic is a well-established genotoxic agent, which has been classified as a class I human carcinogen by IARC [126,127]. Contrary to arsenic, genotoxic properties of antimony are poorly defined and there is no hard evidence of antimony carcinogenic effects in humans [43,44]. Here, we took advantage of *S. cerevisiae* to comprehensively analyze genotoxic properties of Sb(III) using a wide set of genetic, biochemical and DNA damage tests. Using budding yeast as a model organism, we have previously shown that As(III) is able to induce DSBs independently from oxidative stress and replication suggesting that the mechanisms of As(III) genotoxicity are more complex than previously thought [50]. In contrast, it is generally believed that both As(III) and Sb(III) induce DNA damage indirectly by elevating production of ROS and interfering with antioxidant defense systems, which result in oxidative DNA damage including SSBs and replication-associated DSBs [44,126,127]. Similarly to As(III) [50], we found that Sb(III) is a weak inducer of ROS production (Figure 2A,E) and increases oxidative DNA damage only at a high dose (Figure 3A). Moreover, BER-deficient cells displayed moderately increased sensitivity to Sb(III) (Figure 1), whereas Sb(III)-induced DNA damage detected by the comet assay was not diminished in the presence of antioxidant agent (Figure 3B,C). Nevertheless, mutants with impaired antioxidant systems were more sensitive to Sb(III) compared to the wild-type strain (Figure 2D). This is in line of the notion that wild-type yeast cells or whole animals having robust antioxidant systems are well protected from oxidative damage elicited by metals and metalloids [44,128].

In budding yeast, As(III) was shown to trigger DSBs independently from replication and oxidative stress [50]. Unexpectedly, we failed to detect Sb(III)-induced DSBs even at extremely high concentrations (Figure 3D). However, we collected indirect evidence suggesting that Sb(III) may be able to generate replication and oxidative-independent DSBs. DNA damage response (DDR) is activated by DSB-inducing factors in all phases of the cell cycle [82], whereas subtoxic concentrations of H_2_O_2_ and MMS trigger DDR activation exclusively in S phase by generating ssDNA regions and DNA breaks as a result of perturbed replication [83,84]. Similarly to As(III) [50], low doses of Sb(III) activated DDR in S and G2/M phases (Figure 4). In addition, Sb(III)-treated cells synchronized in G2/M exhibited higher incidence of oxidative stress-independent Rad52 nuclear foci (Figure 3B), which serve as a physiological indicator of DNA damage repaired by HR [75]. However, it is important to note that Sb(III)-induced DNA lesions of G2/M cells may represent not only DSBs but also unprotected telomeres (see below). In the absence of BER, H_2_O_2_ and MMS-induced damage can be converted to DNA lesions triggering DDR activation outside S phase [50,83,85]. This was not observed in the BER-deficient mutant synchronized in G1 and exposed to low doses of As(III) or Sb(III) [50] (Figure 6A), consistent with the lack of increased levels of oxidative DNA damage. In contrast, both As(III) and Sb(III) activated DDR in G1 cells lacking the yKU complex, which protects DSBs from resection and thus inhibits robust DDR activation in G1 phase [100,101,102]. This was accompanied by DSB resection manifested by formation of nuclear Rfa1-YFP foci (Figure 6C) and DDR-dependent G1 checkpoint delay (Figure 6D). Importantly, unprotected telomeres do not trigger DNA damage checkpoint activation in G1, even in the absence of yKu [109,129]. Thus, DNA lesions activating DDR in G1-arrested *yku70*Δ cells likely represent Sb(III)-induced DSBs. Consistent with fact that DSBs are predominantly repaired by HR in budding yeast [56], mutants deficient for recombination proteins (*rad51*Δ, *rad52*Δ, *rad59*Δ) or nucleases catalyzing resection of DSB ends (*exo1*Δ *sgs1*Δ) exhibited high sensitivity to Sb(III) (Figure 1).

We also showed that Sb(III) causes perturbation of DNA synthesis leading to formation of ssDNA regions, which may correspond to stalled replication forks and/or ssDNA gaps formed behind the fork as a result of damage bypass (Figure 3E or Figure 5B). Post-replication repair of ssDNA gaps requires two ubiquitin ligases Rad18 and Rad5 as well as HR proteins [54,55]. Consistently, *rad18*Δ and *rad5*Δ mutants were more sensitive to Sb(III) compared to wild-type (Figure 1). Replication fork progression can be blocked by protein complexes trapped on DNA [130]. Interestingly, it has been reported that As(III)-induced oxidative damage leads to the formation of topoisomerase I-DNA complexes [122]. Topoisomerase I relaxes transcription and replication-produced DNA supercoiling by forming transient covalent DNA cleavage complexes [131]. Here, we showed that deletion of *TOP1* in yeast cells attenuates Sb(III)-induced DNA damage (Figure 9B,C) and partially suppresses Sb(III) sensitivity of cells lacking Tel1 kinase (Figure 9A), which is involved in protection of reversed replication forks induced by Top1 poison CPT [121]. Interestingly, induction of the topoisomerase cleavage complexes was also reported in human T cells treated with inorganic As(III) [132]. Montaudon et al. [133] has demonstrated that in contrast to CPT, which delays re-ligation of the break resulting in trapping Top1 on DNA, thiol-reacting agents such as *N*-ethylmaleimide and phenylarsine oxide inhibit DNA cleavage activity of human Top1 by binding to two vicinal cysteines in a highly conserved region. Sb(III) readily binds to sulfhydryl groups in proteins and is tempting to speculate that Sb(III) acts as an inhibitor of Top1 activity by interaction with critical cysteine residues. Given that Top1 is involved in relieving DNA torsional strain during both replication and transcription, a subset of replication-independent DNA damage triggered by Sb(III) may also depend on the Top1 cleavage complex.

Inhibition of DNA repair is a well-established mechanism of As(III) genotoxicity, which involves downregulation of the DNA repair gene expression, disrupting catalytic activity and post-translational modifications of DNA repair proteins as well as their recruitment to the sites of DNA damage [127,134]. There are also a few reports indicating Sb(III) interference with DNA repair pathways [27,47,48]. It has been demonstrated that Sb(III) inhibits removal of UVC-induced cyclobutane pyrimidine dimers by NER due to the decreased expression of the XPE protein, which is specifically involved in the removal of the pyrimidine dimers, and Zn(II) release from the zinc finger domain of the XPA protein resulting in reduced recruitment of XPA to the sites of damage [27]. Sb(III) has also been shown to inhibit the repair of ionizing radiation-induced DSBs in cultured mammalian cells [47,48]. Perturbation of DSB repair by Sb(III) was manifested by diminished recruitment of BRCA1 and RAD51 to DSBs for recombination repair [48]. Interestingly, it has been reported that As(III) impairs the recruitment of BRCA1 and RAD51 to the sites of DNA damage by inhibiting the RNF20-RNF40 E3 ubiquitin ligase-mediated ubiquitination of histone H2B at Lys120 [135]. Inhibition of RNF20-RNF40 involves As(III) binding to critical cysteine residues in the RING finger motif [135]. Here, we demonstrated that the repair of PM-induced DSBs is also impaired by Sb(III) in *S. cerevisiae* (Figure 10C). The H2B-K120Ub-RNF20-RNF40-BRCA1 pathway is not present in budding yeast but several RING finger ubiquitin ligases are involved in the *S. cerevisiae* DNA damage signaling and repair and might be targeted by Sb(III). In addition, we found that Sb(III) distorts actin cytoskeleton (Figure 11A). Inhibition of actin polymerization has been shown to reduce efficiency of DSB repair by HR [125]. Thus, Sb(III) may disturb the repair of DSBs also by targeting actin cytoskeleton.

Additionally, we showed that Sb(III) inhibits rejoining of linear plasmid ends and telomere fusions catalyzed by NHEJ (Figure 8B or Figure 10A). The key NHEJ enzyme is the DNA ligase IV complex composed of the catalytic subunit Dnl4 ligase and the accessory protein Lif1, which stabilizes the enzyme [57]. Structural studies of XRCC4, a human homolog of Lif1, revealed that the C-terminal region involved in XRCC4 homodimer formation contains two vicinal cysteine residues (Cys128 and Cys130), of which Cys130 was suggested to form an inter-chain disulfide bond between monomers [136]. Cys128 and Cys130 are not conserved in the yeast Lif1, although two vicinal cysteine residues (Cys167 and Cys168) corresponding to Ala135 and Glu136 in XRCC4 are present in the dimer interface of Lif1. It is tempting to speculate that Sb(III) may disrupt formation and stability of the Dnl4-Lif1 complex by binding to cysteine residues located in the Lif1 dimer interface.

Finally, we collected genetic data suggesting that Sb(III) causes telomere dysfunction. Mutants defective in telomere maintenance were highly sensitive to Sb(III) (Figure 7). Based on the observation that Sb(III) sensitivity of these mutants could be suppressed by deletion of genes involved in resection of telomere ends or DNA damage checkpoint activation, we inferred that Sb(III) disrupts telomere protection, that is particularly evident in cells with already compromised telomere integrity. Sb(III) treatment had no effect on telomere length (Figure 8A) but led to increased accumulation of the Cdc13 protein on telomeres (Figure 8C,D). Since the Cdc13-Stn1-Ten1 complex preferentially binds to single-stranded telomeric repeats [62,103], higher levels of the Cdc13 protein on the telomeric chromatin suggests generation of longer tracts of single-stranded G-tails at the chromosome ends, which may contribute to Mec1-dependent checkpoint activation in G2/M observed during Sb(III) exposure (Figure 4). Interestingly, it has been shown that As(III) triggers telomere dysfunction in cultured mammalian cells [132,137,138]. Cheng et al. [138] demonstrated that glioma cells treated with arsenic trioxide experienced telomere-associated DDR activation, reduction of the G-tail length without shortening the total length of telomeres and telomere fusions, which were associated with translocation of telomerase from the nucleus to the cytoplasm. The similar mechanism of As(III)-induced impairement of telomere integrity was observed in prostate cancer cells [137]. Interestingly, the prostate cell line PC-3 exhibiting the shortest telomeres among several cancer cell lines tested showed the highest sensitivity to As(III) [137]. The recent study on the effect of As(III) on human CD4 T cells has revealed that As(III) impairs telomere homeostasis not only by telomerase displacement but also by downregulating expression of the telomere sheltering protein TRF2 [132]. It would be interesting to investigate whether Sb(III) causes telomere dysfunction also in mammalian and *Leishmania* cells and what is the molecular mechanism of Sb(III)-induced telomere instability.

Taking into account the complex mechanisms of Sb(III) genotoxicity, we favor the hypothesis that Sb(III) acts as an indirect genotoxin by interfering with activity, folding, protein-protein interactions and/or subcellular localization of proteins involved in DNA metabolism and antioxidant defense systems. In the future, it will be interesting to determine molecular targets of Sb(III) using proteomic approaches. Our results also indicate that further research into the mechanisms of antimony genotoxicity should be conducted in animals to conclusively establish whether antimony is carcinogenic or may potentiate carcinogenicity of other compounds. In addition, better understanding of antimony genotoxic properties could also be used in the development of new strategies for treating tropical diseases caused by protozoa as well as in anti-cancer therapies.

## 4. Materials and Methods

### 4.1. Yeast Strains and Growth Conditions

Yeast strains used in this work are listed in Supplementary Table S1. Deletion mutants were constructed using either a PCR-based replacement method [139] or by genetic crossing of relevant mutants followed by tetrad dissection. To investigate the sensitivity of yeast strains to genotoxins, mid-log cultures were 10-fold serially diluted and spotted on solid YPD media containing various concentrations of Sb(III), metals or DNA damaging agents.

### 4.2. Intracellular ROS Measurements

To assess intracellular levels of ROS, yeast cultures were pretreated with 5 µg/mL of DHR123 for 15 min and then exposed to Sb(III), menadione or H_2_O_2_ for 2 h. Next, aliquots of cells were taken and immediately analyzed by flow cytometry using Guava^®^ easyCyte (Merck, Darmstadt, Germany) to measure levels of green fluorescence of R123 formed after oxidation of DHR123 by ROS. Untreated samples were used as a control of autofluorescence level.

### 4.3. The 2,3,5-triphenyltetrazolium Chloride (TTC) Assay

Yeast cells were grown in minimal medium to mid-log phase and then treated or not with Sb(III) for 2 h. Equal amounts of cells were centrifuged, washed with water, resuspended in minimal medium containing 0.1% TTC and incubated for 2 h at 30 °C in the dark. Next, cells were centrifuged, washed with dimethyl sulfoxide and aliquots were transferred to 96-well plate to measure the absorbance at λ = 485 nm using a Multiscan GO microplate spectrophotometer (Thermo Fisher Scientific, Waltham, MA, USA).

### 4.4. Measurements of 8-hydroxy-2′-deoxyguanosine (8-OHdG) Levels

Genomic DNA from yeast cells was isolated using GeneMATRIX UNIVERSAL DNA/RNA/Protein Purification Kit (EURx, Gdansk, Poland). Oxidative DNA damage in the form of 8-OHdG was quantified using OxiSelect™ Oxidative DNA Damage ELISA Kit (Cell Biolabs, San Diego, CA, USA) according to the manufacturer’s instructions and a Multiscan GO microplate spectrophotometer (Thermo Fisher Scientific, Waltham, MA, USA).

### 4.5. Fluorescence Microscopy

Analysis of cellular localization of fluorescently labelled Rfa1, Rad52 and Tub1 proteins and DNA staining with 4′,6-diamidino-2-phenylindole were performed in live cells using an Axio Imager M1 epifluorescence microscope (Carl Zeiss, Oberkochen, Germany) equipped with a 100× immersion oil objective (Plan-Neofluar 1006/1.30), a GFP and RFP filter set and DIC. Images were collected using an AxioCam MRc digital color camera and processed with AxioVision 4.5 software (Carl Zeiss, Oberkochen, Germany. To visualize actin fibers cells were fixed with formaldehyde (3.7% final) for 2 h, washed with water and PBS followed by incubation with 0.2% Triton-X for 10 min. After several washes with PBS, cells were incubated in the presence of 1.5 µM rhodamine-phalloidin in the dark for 1 h at 4 °C. Finally, cells were washed with PBS and analyzed with epifluorescence microscope.

### 4.6. Yeast Alkaline Comet Assay

Approximately 10^5^ cells were harvested by centrifugation and washed with S-buffer (1 M sorbitol, 25 mM KH_2_PO_4_, pH 6.5). Then, cells were resuspended in 1.5% low melting agarose dissolved in S buffer containing 2 mg/mL Zymolyase 20T. Subsequently, 40 μL of cell suspension was spread on microscope slides, overlaid with 1% agarose and covered with coverslips followed by 45 min incubation at 30 °C to degrade yeast cell wall. Next, slides were incubated for 10 min at 4 °C and coverslips were removed. In the following step, slides were incubated in alkaline lysis solution (30 mM NaOH, 1 M NaCl, 50 mM EDTA, 10 mM Tris-HCl, pH 10) for 20 min at 4 °C and then washed four times in electrophoresis buffer (30 mM NaOH, 10 mM EDTA, 10 mM Tris-HCl, pH 10). DNA electrophoresis was conducted in electrophoresis buffer at 4 °C for 20 min at 25 V. After electrophoresis, slides were incubated in 10 mM Tris-HCl (pH 7.4) for 10 min, followed by sequential incubation in 76% and 95% ethanol for 5 min. Finally, slides were left to air-dried for 15 min, stained with SYTOX Green and observed with Axio Imager M1 epifluorescence microscope (Carl Zeiss, Oberkochen, Germany). Comet tail lengths were analyzed using CometScore 2.0 software (TriTek, Sumerduck, VA, USA).

### 4.7. Pulsed Field Gel Electrophoresis (PFGE)

Preparation of agarose-embedded chromosome DNA and PFGE were performed as previously described [50].

### 4.8. Chromatin Endogenous Cleavage (ChEC)

ChEC analysis was performed as previously described [72]. Briefly, about 10^8^ cells were arrested with 0.1% sodium azide and then permeabilized with 1% digitonin for 5 min at 30 °C. Then, cells were incubated with 2 mM of CaCl_2_ at 30 °C for 45 min to activate micrococcal nuclease. Next, cell wall was degraded with 2 mg/mL Zymolyase 20T and DNA was isolated by ethanol precipitation and phenol:chloroform:isoamyl alcohol purification followed by RNA digestion with 0.25 μg/mL RNAse. Equal concentrations of total DNA were loaded and resolved on 1.2% 1 × Tris-borate-EDTA (TBE) agarose gel and stained with ethidium bromide.

### 4.9. Western Blot Analysis

To isolate total protein extracts ~2 × 10^7^ of yeast cells were resuspended in 100 µl of lysis buffer (2 M NaOH, 7% β-mercaptoethanol) followed by addition of 100 µl of 50% trichloroacetic acid. After centrifugation, protein pellet was rinsed with 100 µl of 1 M Tris (pH 8) and resuspended in 40 µl of Laemmli buffer. Proteins were resolved on 8% (for detection of Rad53) or 15% (for detection of H2A and H2A S129 phosphorylation) SDS-PAGE, blotted on nitrocellulose membranes and probed with anti-Rad53 (ab104232, Abcam, Cambridge, UK), anti-phospho H2A S129 (ab15083, Abcam, Cambridge, UK) or anti-H2A (07-146, Merck, Darmstadt, Germany) antibodies. Chemiluminescence signal detection was performed using the Bio-Rad ChemiDoc MP System and Image Lab software (Bio-Rad, Hercules, CA, USA).

### 4.10. Cell Cycle Analysis

To arrest yeast cells in G1 phase, cells were incubated with 5 μM α-factor for 2 h. To synchronize yeast cells at the G2/M boundary, cells were treated with 15 μg/mL nocodazole for 2 h. To analyze the cell cycle progression of yeast cells, flow cytometry analysis of DNA content was performed as follows. At indicated time points, 500 µl of yeast cultures were fixed with 70% ethanol and stored at 4 °C. Then, cells were extensively washed with water, digested for 2 h with 0.25 μg/mL RNase at 50 °C and subsequently incubated with 1 μg/mL pepsin at 37 °C. Next, cells were sonicated, stained with 2.5 μM SYTOX Green for 30 min and then analyzed by flow cytometry using Guava^®^ easyCyte (Merck, Darmstadt, Germany). To determine the fraction of cells arrested in G1, the α-factor-nocodazole trap assay was performed as described previously [95]. To assess duration of G2/M checkpoint arrest, cells were fixed with ethanol, stained with DAPI and then observed with epifluorescence microscope to score the percentage of binucleate large-budded cells.

### 4.11. Analysis of Telomere Length

To isolate genomic DNA for analysis of telomere length ~10^8^ of log-phase yeast cells were resuspended in lysis buffer (100 mM NaCl, 2% Triton X, 1% SDS, 1 mM EDTA, 1 mM Tris, pH 8.0) followed by addition of phenol:chloroform-isoamyl alcohol. Next, cells were lysed with glass beads in a bead beater at 4 °C. After centrifugation, DNA was precipitated with 96% ethanol, air dried and resuspended in water. 10 µg of genomic DNA was digested with *Xho*I restriction enzyme and then separated on 0.9% agarose gel at 45 V for ~20 h. After electrophoresis DNA was transferred to positively charged nylon membrane by capillary transfer in 10 × saline-sodium citrate (SCC) buffer, overnight. Next day membrane was UV crosslinked followed by Southern blot analysis. Southern blot was performed using DIG-High Prime DNA Labeling and Detection Starter Kit II (Merck, Darmstadt, Germany) following manufacturer’s instructions. The telomere-specific probe was obtained by digestion of pCT300 plasmid with the *EcoR*I restriction enzyme. Chemiluminescence signal detection was performed using the Bio-Rad ChemiDoc MP System and Image Lab software (Bio-Rad, Hercules, CA, USA).

### 4.12. Detection of Telomere Fusions

To isolate genomic DNA for analysis of telomere fusions, ~10^9^ of stationary phase yeast cells were resuspended in Tris-EDTA (TE) buffer containing 1% SDS followed by addition of phenol:chloroform-isoamyl alcohol. Next, cells were lysed with glass beads in a bead beater at 4 °C and lysates were centrifuged. Supernatants were transferred to fresh tubes, mixed with 2 volumes of 96% ethanol and incubated for 1 min in liquid nitrogen. After centrifugation, pellets were resuspended in TE buffer followed by addition of 1 volume of 96% ethanol and another incubation in liquid nitrogen for 1 min. Next, pellets were spun, rinsed with 70% ethanol, centrifuged again, air dried and resuspended in water. Amplification of telomere-telomere fusions by PCR was performed using CloneAmp HiFI PCR premix (Takara Bio, Kusatsu, Japan), 1 µM of Y’ and X2 primers (Supplementary Table S2) and 50 ng of genomic DNA. Amplification conditions were: 98 °C for 1 min then 40 cycles of 98 °C for 10 s, 65 °C for 15 s, 72 °C for 10 s, followed by 72 °C for 2 min. PCR products were resolved on 1.2% 1 × TBE agarose gel and stained with ethidium bromide. Fluorescence was analyzed with Bio-Rad ChemiDoc MP System (Bio-Rad, Hercules, CA, USA).

### 4.13. Plasmid Repair Assay

Efficiency of rejoining cohesive DNA ends was determined by the plasmid repair assay as previously described [123]. Briefly, yeast cells were transformed with 400 ng of *Pst*I-linearized or uncut YCplac111 plasmid using the lithium acetate method [140]. In parallel, cultures were exposed to 0.2 mM Sb(III) for 2 h prior transformation procedure. After transformation, diluted samples were plated onto minimal selective media and colonies were counted after incubation at 30 °C for 4 days. In addition, appropriate dilutions of transformed cells were plated on rich media to determine CFU. Transformation efficiency was expressed as the ratio of colonies obtained with the linear plasmid divided by colonies obtained with the uncut plasmid and then divided by the number of viable cells used for transformation.

### 4.14. Chromatin Immunoprecipitation-Quantitative PCR (ChIP-qPCR)

ChIP-qPCR of Cdc13-cMyc was performed as previously described [141]. Immunoprecipitation reaction was performed using 4 µL of anti-cMyc antibodies (9E10, Roche, Basel, Switzerland). Primers used for qPCR are listed in Supplementary Table S2.

## Data Availability

Data sharing not applicable.

## Supplementary Materials

The following are available online at https://www.mdpi.com/article/10.3390/ijms22094510/s1, Figure S1: Quantification of DNA digestion shown in Figure 3E, Figure S2: Source data for western blots shown in Figure 4, Figure S3: Source data for western blots shown in Figure 6A,B, Figure S4: The growth of cdc13-1 cells in the presence of various toxins, Figure S5: Quantification of DNA digestion shown in Figure 9C, Table S1: Yeast strains used in this work, Table S2: Oligonucleotides used in this work.

## Abbreviations

IARC	International Agency for Research on Cancer
ROS	Reactive oxygen species
GSH	Glutathione
TSH	Trypanothione
DSB	Double-strand break
HR	Homologous recombination
NER	Nucleotide excision repair
ssDNA	Single-stranded DNA
DDR	DNA damage response
BER	Base excision repair
DDT	DNA damage tolerance
NHEJ	Non-homologous end joining
AP	Apurinic/apyrimidinic
TS	Template switch
SSA	Single-strand annealing
BIR	Break-induced replication
R123	Rhodamine 123
DHR123	Dihydrorhodamine 123
TTC	Triphenyltetrazolium chloride
8-OHdG	8-hydroxy-2′-deoxyguanosine
SSB	Single-stranded DNA break
PFGE	Pulsed field gel electrophoresis
ChEC	Chromatin endogenous cleavage
MN	Micrococcal nuclease
MMS	Methyl methanesulfonate
ChIP-qPCR	Chromatin immunoprecipitation-quantitative PCR
PM	Phleomycin
CPT	Camptothecin
CFU	Colony forming units
DIC	Differential interference contrast
