# Supplementary material for: Complex Mechanisms of Antimony Genotoxicity in Budding Yeast Involves Replication and Topoisomerase I-Associated DNA Lesions, Telomere Dysfunction and Inhibition of DNA Repair
Source: Int J Mol Sci. 2021 Apr 26;22(9):4510. doi: 10.3390/ijms22094510 (PMC8123508; doi:10.3390/ijms22094510)
Supplement: Supplementary file 1 [file ijms-22-04510-s001.zip › ijms-1175777-supplementary.pdf]

Supplementary Materials

Supplementary Figures S1–S5

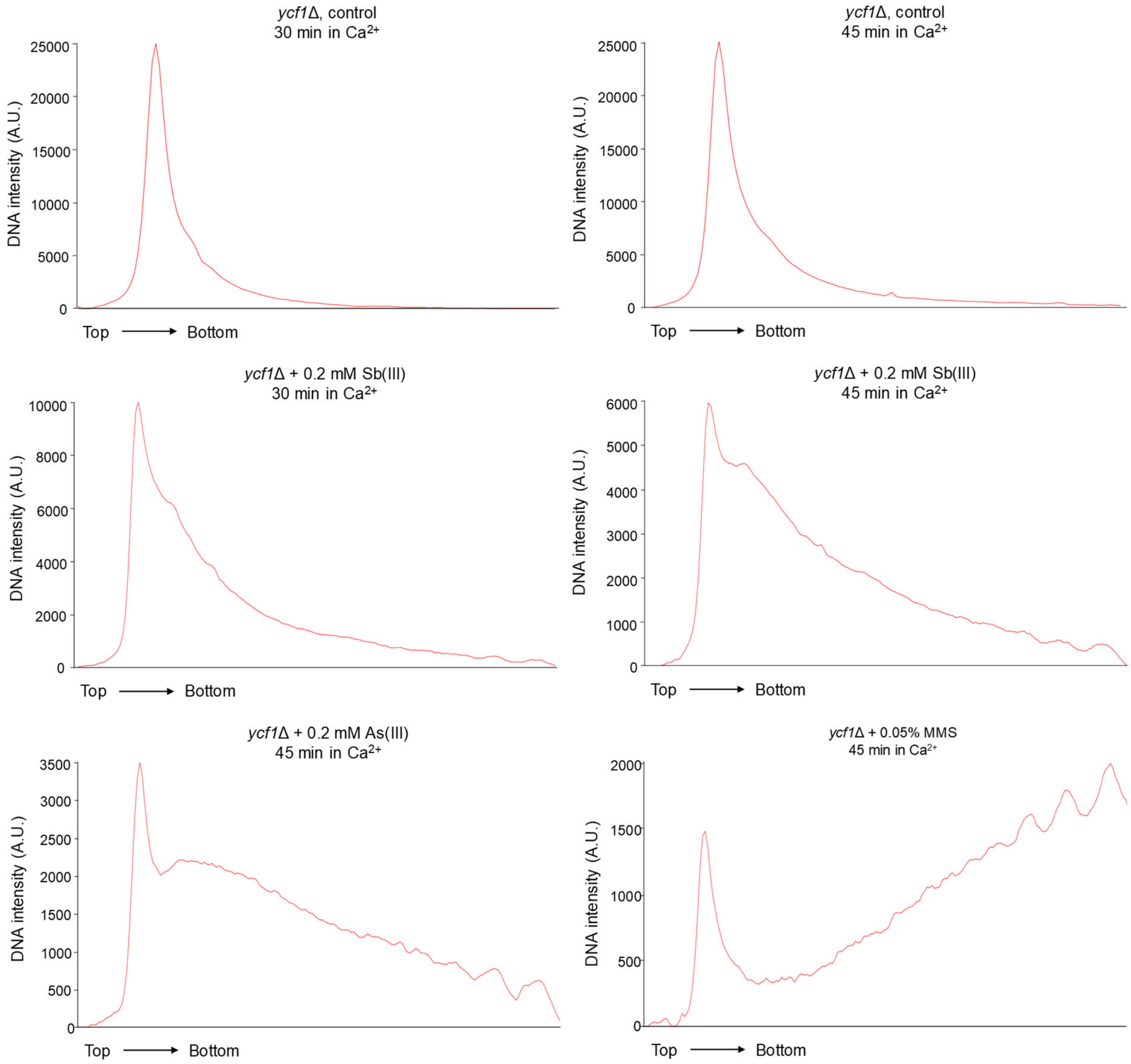

Supplementary Figure S1. Quantification of DNA digestion shown in Figure 3E.

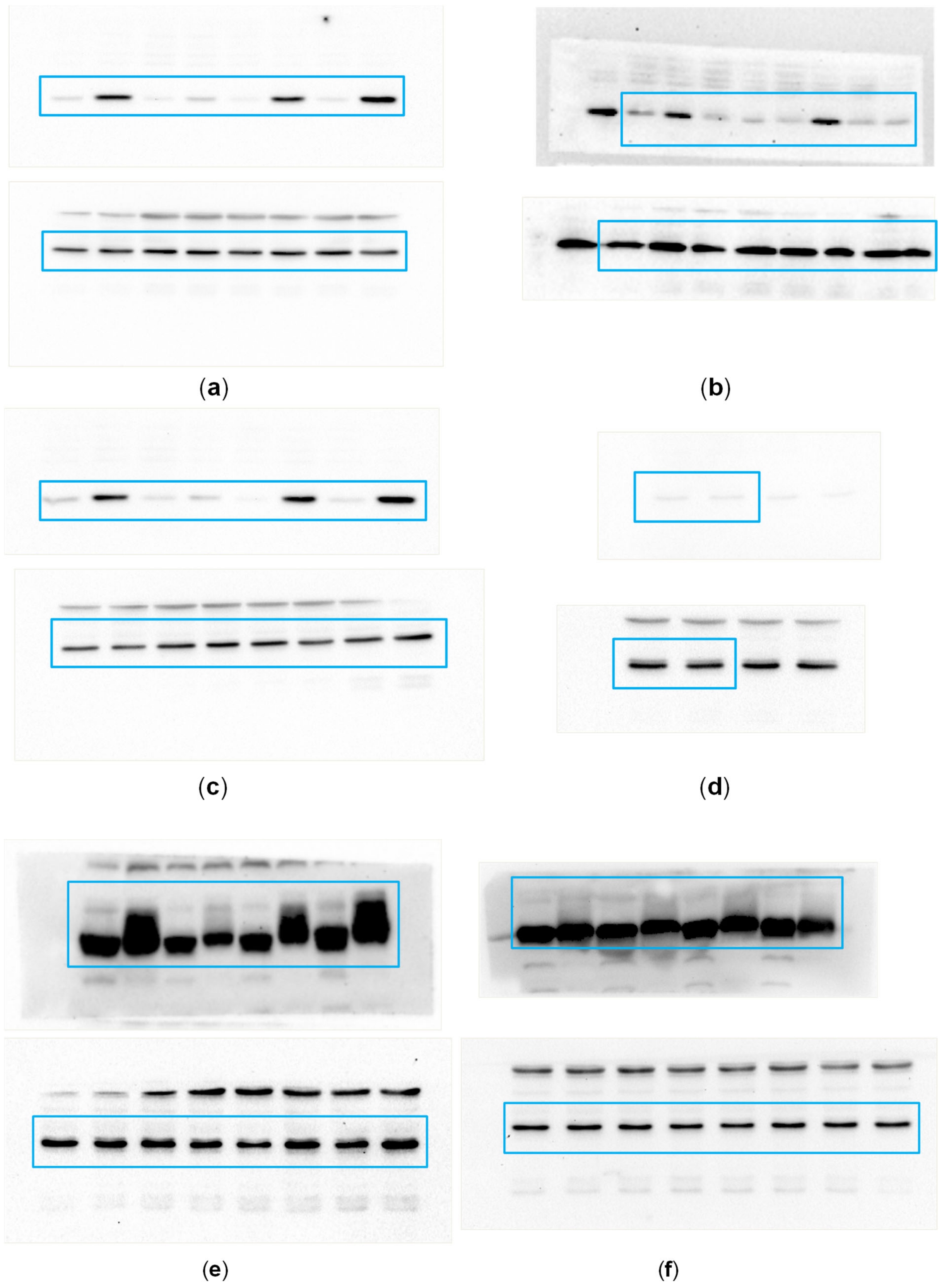

Supplementary Figure S2. Source data for western blots shown in Figure 4.

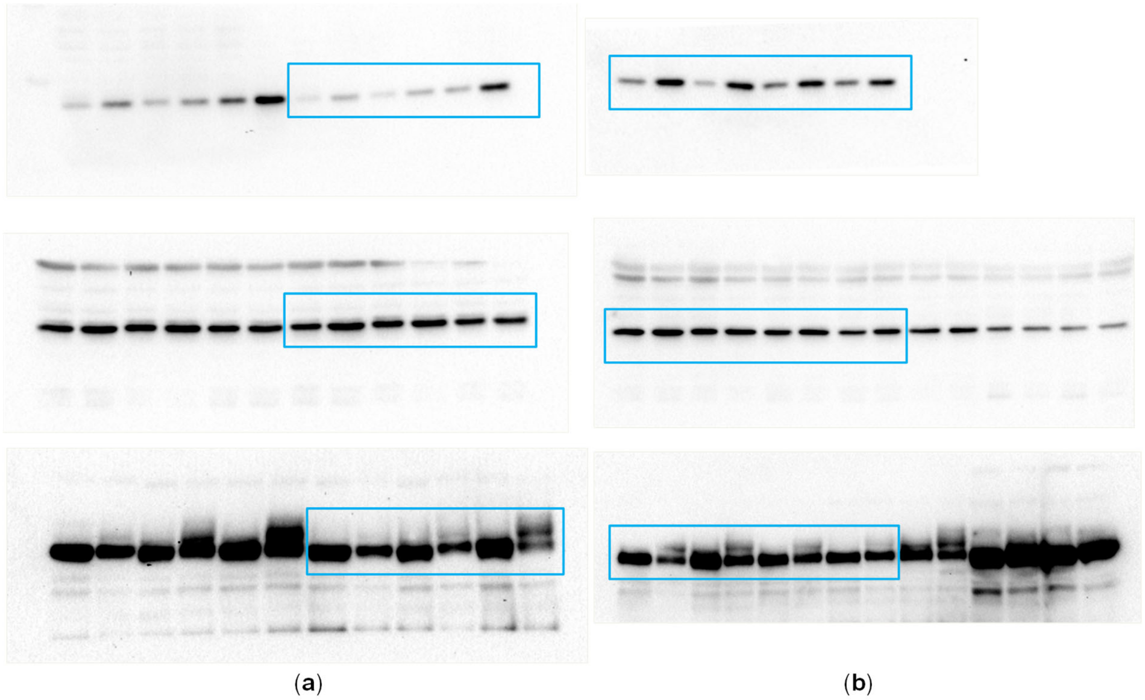

Supplementary Figure S3. Source data for western blots shown in Figure 6A and 6B.

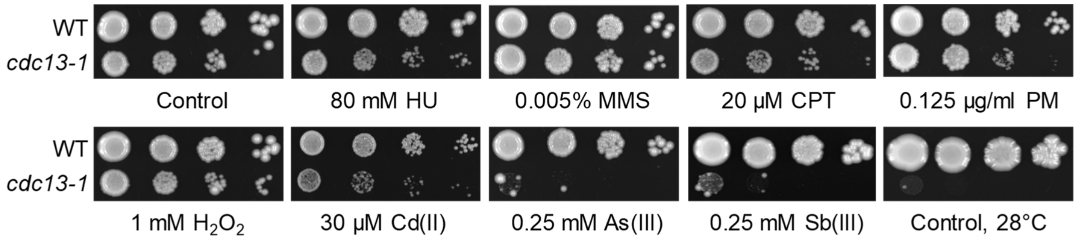

Supplementary Figure S4. The growth of *cdc13-1* cells in the presence of various toxins. Exponentially growing cell cultures were serially diluted, spotted onto YPD plates with or without indicated compounds and incubated at 25 °C (or 28 °C) for 3 days.

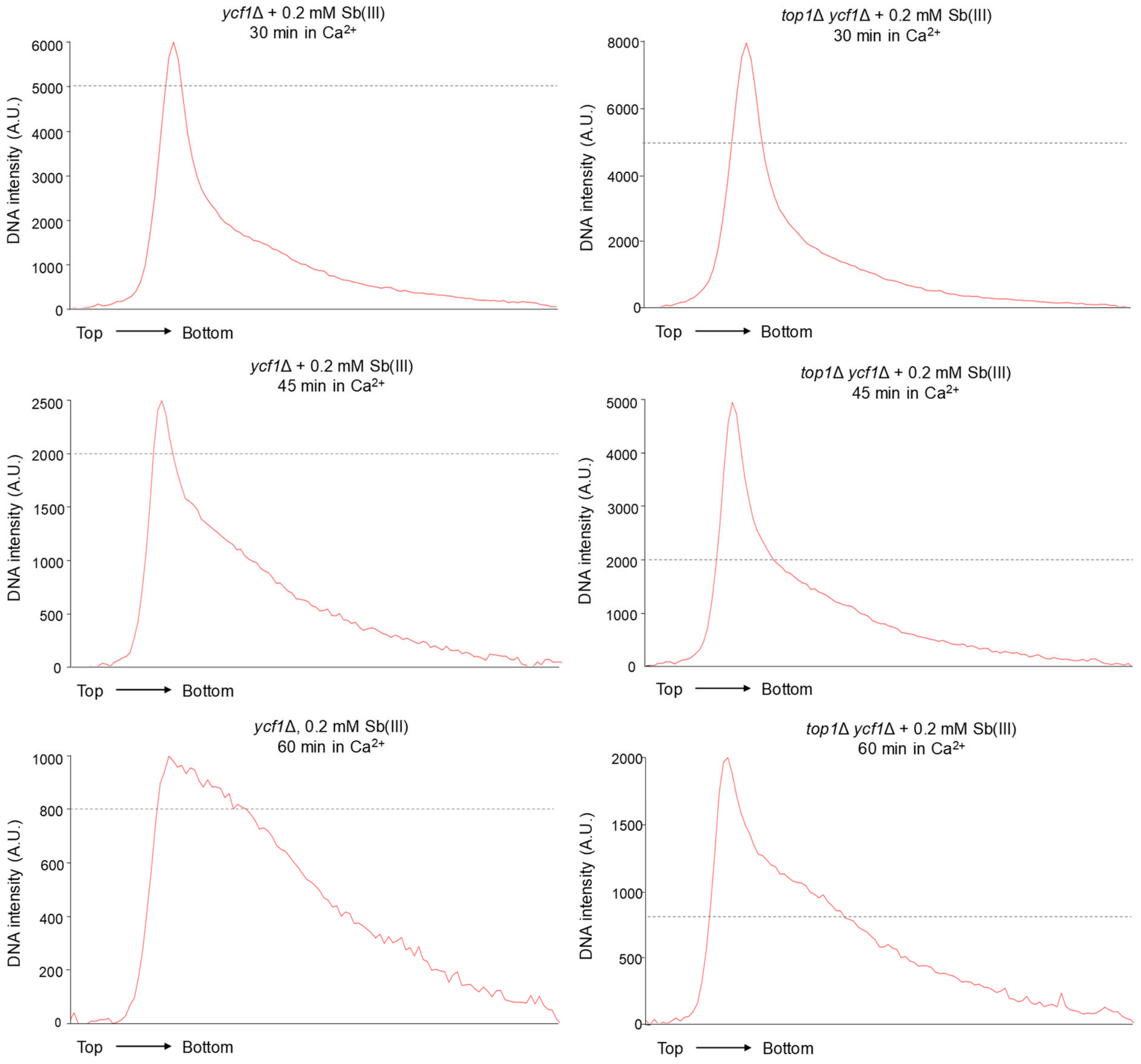

Supplementary Figure S5. Quantification of DNA digestion shown in Figure 9C.

Supplementary Tables S1–S2

Supplementary Table S1. Yeast strains used in this work.

Strain	Genotype	Source
W303-1a	*MAT***a** *ade2-1 can1-100 ura3-1 his3-11,15 leu2-3,112 trp1-1 RAD5*	R. Rothstein
SM011	W303-1a, *ycf1Δ:: natMX4*	This study
SM012	W303-1a, *ycf1Δ::natMX4 apn1Δ::TRP1 apn2Δ::kanMX6*	This study
SM013	W303-1a, *ycf1Δ::natMX4 rad14Δ::kanMX6*	This study
SM014	W303-1a, *ycf1Δ::loxP- kanMX-loxP rad18Δ::natMX4*	This study
SM015	W303-1a, *ycf1Δ::natMX4 rad5Δ::HIS3*	This study
SM016	W303-1a, *ycf1Δ::natMX4 dnl4Δ::kanMX6*	This study
SM017	W303-1a, *ycf1Δ::natMX4 yku70Δ::kanMX6*	This study
SM018	W303-1a, *ycf1Δ::natMX4 rad52Δ::kanMX6*	This study
SM019	W303-1a, *ycf1Δ::natMX4 rad51Δ::kanMX6*	This study
SM020	W303-1a, *ycf1Δ::natMX4 rad59Δ::kanMX6*	This study
SM021	W303-1a, *ycf1Δ::natMX4 exo1Δ::kanMX6*	This study
SM022	W303-1a, *ycf1Δ::natMX4 sgs1Δ:: LEU2*	This study
SM023	W303-1a, *ycf1Δ::natMX4 exo1Δ::kanMX6 sgs1Δ:: LEU2*	This study
SM024	W303-1a, *ycf1Δ::natMX4 mec1Δ::TRP1 sml1Δ::HIS3*	This study
SM025	W303-1a, *ycf1Δ::natMX4 tel1Δ::URA3*	This study
SM026	W303-1a, *ycf1Δ::natMX4 mec1Δ::TRP1 sml1Δ::HIS3 tel1Δ::URA3*	This study
SM027	W303-1a, *ycf1Δ::natMX4 rad9Δ::kanMX6*	This study
SM028	W303-1a, *ycf1Δ::natMX4 tel1Δ::URA3 yku70Δ::kanMX6*	This study
SM029	W303-1a, *ycf1Δ::natMX4 mec1Δ::TRP1 sml1Δ::HIS3 yku70Δ::kanMX6*	This study
SM030	W303-1a, *ycf1Δ::natMX4 rad9Δ::URA3 yku70Δ::kanMX6*	This study
SM031	W303-1a, *ycf1Δ::natMX4 yku70Δ::kanMX6 tel1Δ::URA3*	This study
SM032	W303-1a, *ycf1Δ::natMX4 pif1Δ::URA3*	This study
DLY1108	W303-1A, *cdc13-1*	D. Lydall
SM033	DLY1108, *ycf1Δ::natMX4*	This study
SM034	DLY1108, *ycf1Δ::natMX4 pif1Δ::URA3*	This study
SM035	DLY1108, *ycf1Δ::natMX4 exo1Δ::kanMX6*	This study
SM036	W303-1a, *ycf1Δ::natMX4 top1Δ::kanMX6*	This study
SM037	W303-1a, *ycf1Δ::natMX4 tel1Δ::URA3 top1Δ::kanMX6*	This study
SM038	W303-1a, *ycf1Δ::natMX4 uls1Δ::TRP1*	This study
W3749-14C	W303-1a, *ADE2, bar1Δ::LEU2, RAD52-YFP*	R. Rothstein
SM039	W303-1A, *ycf1Δ::natMX4 RAD52-YFP*	This study
W3775-12C	W303-1a, *ADE2, bar1Δ::LEU2, RFA1-YFP*	R. Rothstein
SM040	W303-1A, *ycf1Δ::natMX4 RFA1-YFP*	This study
SM041	W303-1A, *ycf1Δ::natMX4 yku70Δ::kanMX6 RFA1-YFP*	This study
BYR52MN	*MAT***a** *his3Δ1 leu2Δ0 ura3Δ0 met15Δ0 RAD52-MN::HIS3MX6*	F. Prado
SM042	BYR52MN, *ycf1Δ::natMX4*	This study
SM043	BYR52MN, *ycf1Δ::natMX4 top1Δ::kanMX6*	This study
SM044	W303-1a, *ycf1Δ::natMX4 GFP-TUB1::URA3*	This study
RW124	W303-1a, *yap1Δ::loxP*	Lab collection
pGAL-SOD1	W303-1A, *pGAL-SOD1::HIS3*	D.A. Knorre
pGAL-SOD2	W303-1A, *pGAL-SOD1::HIS3*	D.A. Knorre
PP529b	W303-1A, *URA3-GPD-TK phENT1-LEU2 bar1Δ::LEU2 CDC13-18Myc-HIS3*	V. Géli
SM045	PP529b, *ycf1Δ::natMX4*	This study

Supplementary Table S2. Oligonucleotides used in this work.

Amplified region	Forward Primer	Reverse Primer
X-Y′ telomeres	TGTGGTGGTGGGATTAGAGTGGTAG	TTAGGGCTATGTAGAAGTGCTG
Telomere VI-R	ATCATTGAGGATCTATAA	CTTCACTCCATTGCG
ARS306	CCCCAATCCCAATAGTTCGA	TGCGCCGCTCATACGA
ACT1	GGCCAAATCGATTCTCAAAA	GCCTTCTACGTTTCCATCCA
